# *triangulaR:* an R package for identifying AIMs and building triangle plots using SNP data from hybrid zones

**DOI:** 10.1038/s41437-025-00760-2

**Published:** 2025-04-12

**Authors:** Ben J. Wiens, Lucas H. DeCicco, Jocelyn P. Colella

**Affiliations:** Biodiversity Institute, Department of Ecology and Evolutionary Biology, University of Kansas, Lawrence, KS 66045 USA

**Keywords:** Population genetics, Bioinformatics, Population genetics, Evolutionary genetics, Speciation

## Abstract

Hybridization provides a window into the speciation process and reshuffles parental alleles to produce novel recombinant genotypes. Presence or absence of specific hybrid classes across a hybrid zone can provide support for various modes of reproductive isolation. Early generation hybrid classes can be distinguished by their combination of hybrid index and interclass heterozygosity, which can be estimated with molecular data. Hybrid index and interclass heterozygosity are routinely calculated for studies of hybrid zones, but available resources for next-generation sequencing datasets are computationally demanding and tools for visualizing triangle plots are lacking. Here, we provide a resource for identifying ancestry-informative markers (AIMs) from single nucleotide polymorphism (SNP) datasets, calculating hybrid index and interclass heterozygosity, and visualizing the relationship as a triangle plot. Our methods are implemented in the R package *triangulaR*. We validate our methods on an empirical dataset and simulations of genetic data from a hybrid zone between two parental groups at low, medium, and high levels of divergence. *triangulaR* provides accurate and precise estimates of hybrid index and interclass heterozygosity with sample sizes as low as five individuals per parental group, and similar levels of error as another program for hybrid index and interclass heterozygosity estimation, *bgchm*. We explore various allele frequency difference thresholds for AIM identification, and how this threshold influences the accuracy and precision of hybrid index and interclass heterozygosity estimates. We contextualize interpretation of triangle plots by describing theoretical expectations under Hardy-Weinberg Equilibrium and provide recommendations for best practices for identifying AIMs and building triangle plots.

## Introduction

Research on hybridization provides insight into the evolution of reproductive isolation, the genetic basis of phenotypic variation, and novel modes of adaptation (Jones et al. 2018; Cronemberger et al. 2020; Aguillon et al. 2021; Nikolakis et al. 2022). Evolutionary outcomes of hybridization often have implications for conservation and management of genetic diversity in natural populations (Chan et al. 2019). A common goal in studies of hybridization is to classify individuals as hybrids or as members of the parental groups (Gompert and Buerkle 2013). Six classes are typically used to describe genetic variation in hybrid zones (Fitzpatrick 2012): the first filial generation (F1), second filial generation (F2), backcrosses in each direction (BC), and the parental groups (P1, P2). Such classification serves as a starting point to describe hybrid zone dynamics and form hypotheses about the genetic, ecological, and environmental mechanisms that shape evolutionary outcomes of hybridization (Simon et al. 2018; Thompson et al. 2023).

**Fig. 1:**
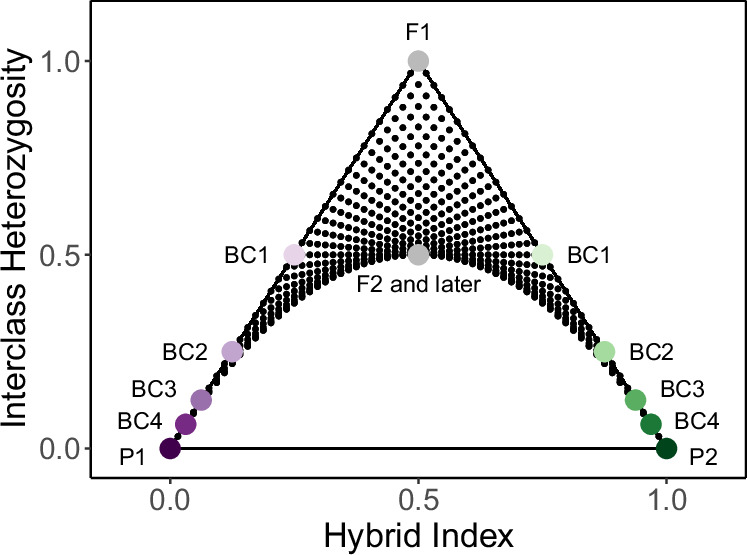
A triangle plot illustrating the theoretical expectations for combinations of hybrid index and interclass heterozygosity under Hardy-Weinberg Equilibrium (HWE). Large, colored points identify parental taxa (P1, P2) and hybrid classes (F1 = first filial generation, F2 = second filial generation, BC = backcross and the number of generations backcrossed toward the nearest parental population). Smaller black points show all possible combinations of hybrid index and interclass heterozygosity after six generations of mixing under HWE.

Molecular data contain the necessary information for assigning hybrid classes (Lynch 1991), which can be done by pairing hybrid index (i.e. ancestry proportions, or the proportion of alleles inherited from each parental group) with interclass heterozygosity (the proportion of loci with alleles from both parental groups). Visualizations of interclass heterozygosity (y-axis) against hybrid index (x-axis) are referred to as triangle plots, because the set of possible coordinates forms the shape of a triangle (Fig. 1). Hybrid index is measured in terms of proportion of ancestry from one parental group, such that individuals belonging to that parental group have a hybrid index of 1 and individuals of the other parental group have a hybrid index of 0. If only fixed differences between the parentals are used to calculate hybrid index, each parental individual, by definition, will have an interclass heterozygosity of 0. By the same logic, F1s will have an interclass heterozygosity of 1 and hybrid index of 0.5, assuming no gene conversion during recombination. If including sites that are not fixed for alternate alleles in the parental groups, but which still show high differentiation, those values may not be matched exactly, but hybrid classes are still identifiable (Rosenberg et al. 2003; Fitzpatrick 2012).

An early method (*introgress*) took a maximum likelihood approach to infer hybrid index and heterozygosity from codominant markers (e.g. amplified fragment length polymorphisms) or biallelic molecular markers (Buerkle 2005; Gompert and Buerkle 2009). More recently, Bayesian methods (*bgc*, Gompert and Buerkle 2012; *ENTROPY*, Shastry et al. 2021; *bgchm*, Gompert et al. 2024) have been developed to infer interpopulation ancestry and admixture proportions from single nucleotide polymorphism (SNP) genotypes and genotype likelihoods. Notably, instead of interclass heterozygosity, *bgchm* uses Bayesian statistics to estimate interpopulation ancestry (Q10). While both statistics describe the proportion of loci with mixed ancestry based on the observed combination of alleles, Q10, as implemented in *bgchm*, takes into account the observed parental allele frequencies to estimate the proportion of loci with ancestry from both parental groups, regardless of allelic state (Gompert et al. 2024). For more on the distinction between interclass heterozygosity and Q10, see Gompert and Buerkle (2013). While those methods provide substantial insight into the evolutionary ecology of hybridization, they can be computationally intensive for large datasets and do not always provide intuitive visualizations of triangle plots. As such, there is a need for computationally simple methods that facilitate quick descriptions of admixture and identification of hybrid classes when exploring SNP datasets.

When the genotypes for many (e.g. thousands) biallelic sites are known with high confidence, a simple analytical approach to ancestry estimation can be taken. With parental individuals included in the sample, ancestry-informative markers (AIMs) can be identified and used to calculate hybrid index and interclass heterozygosity. Whole genomes and reduced-representation sequencing methods (e.g. RADseq, target capture) can provide thousands of SNPs from which AIMs can be identified, but practices for sampling parental populations and identifying AIMs vary widely (e.g. Del-Rio et al. 2022; Ocampo et al. 2023; Preckler-Quisquater et al. 2023). The sample size needed for each parental group is an important consideration because any method for identifying AIMs depends on identifying genomic sites that reliably show differentiation between the parental groups (Rosenberg et al. 2003). Short of sampling all individuals in a population, it is impossible to know the true allele frequencies. Therefore, sample sizes must be large enough to provide reasonable estimates of allele frequencies in each parental population, while balancing the expense of sampling and sequencing many individuals.

Another important consideration is the method used to identify AIMs. Intuitively, SNPs with fixed differences are most informative for estimating ancestry proportions, but for some genomic datasets restricting AIMs to such sites is not feasible (DeRaad et al. 2023). When there are not enough fixed differences between two parental groups, a lower threshold of differentiation must be used to identify AIMs. Therefore, it is worthwhile to explore the consequences of lower allele frequency difference thresholds for identifying AIMs, building triangle plots, and distinguishing among hybrid classes.

Here, we introduce the R package *triangulaR* (https://github.com/omys-omics/triangulaR), which provides simple and quick calculations of hybrid index and interclass heterozygosity from SNP datasets to build triangle plots and classify hybrids. To validate these methods, we simulated genetic data for hybrid zones with parental groups at various levels of divergence. We also tested *triangulaR* on an empirical RADseq dataset from a hybrid zone between two closely related species of *Passerella* sparrows in south-central Alaska. Based on our simulations, we find that sample sizes as low as five individuals per parental group provide reliable estimates of hybrid index and interclass heterozygosity, even when the parental groups are minimally divergent. We also show that when divergence is minimal, there is a practical tradeoff between restricting AIMs to few, highly informative sites versus relaxing the allele frequency difference threshold to include more, but less informative sites. We outline best practices for sampling parental populations, identifying AIMs, and building triangle plots to identify hybrids and hybrid classes from molecular datasets.

## Methods

### Package description

When parental populations are included in a sample from a hybrid zone and SNP genotypes are known with high confidence, hybrid index and interclass heterozygosity can be calculated analytically. Specifically, those metrics can be calculated by identifying AIMs, defined as sites with an allele frequency difference (δ) between the parental groups that is above a chosen threshold, and then polarizing alleles at those sites to determine in which parental group each allele has a higher frequency (Rosenberg et al. 2003).

This approach is implemented in the R package *triangulaR*. The main functions of this package are outlined in Supplementary Table S1, and package documentation and a detailed tutorial are available at https://github.com/omys-omics/triangulaR. Briefly, *triangulaR* accepts as input genotype data for biallelic SNPs and leverages the functionality of the R package *vcfR* to read in VCF files and store vcfR objects (Knaus and Grünwald 2017). The only other required input is an R dataframe with individual identifiers in the first column and population assignments in the second column. Hybrid index and interclass heterozygosity are calculated in two steps. First, SNPs with an allele frequency difference between the parental groups above a user-defined threshold are identified as AIMs, and a vcfR object containing individual genotypes at only these SNPs is returned. An allele frequency difference of 1 indicates a fixed difference between the two parental populations. In the second step, that vcfR object is used for calculating hybrid indices, which is done by summing the number of alleles in each individual that match one parental group and dividing by the total number of nonmissing sites present for that individual. Average interclass heterozygosity is also calculated during that step by counting the number of observed heterozygous sites and dividing by the total number of nonmissing sites. Hybrid index and interclass heterozygosity estimates are returned along with user-defined population assignment and percent missing data for each individual, formatted in an R dataframe. Results can be visualized with a wrapper function (*triangle.plot*) that uses the R package *ggplot2* (Wickham 2011) to plot the observed hybrid index and interclass heterozygosity values for each individual and draw the outline of the possible space on a triangle plot under Hardy-Weinberg Equilibrium (HWE). Results can be colored by pre-defined populations or percent missing data to aid interpretation.

### Possible space on triangle plots under HWE

Triangle plots are used for identifying hybrid classes (e.g. F1s, backcrosses, etc.) and inferring the presence and strength of barriers to reproduction (Christe et al. 2016; Fitzpatrick 2012; Lindtke et al. 2012; Pulido-Santacruz et al. 2018). It is therefore useful to consider the possible space on a triangle plot under HWE. By this, we mean the possible combinations of hybrid index and interclass heterozygosity in the generations following interbreeding between two distinct parental groups, assuming random mating and the absence of selection, drift, or new mutations. Describing this space provides neutral expectations for the possible combinations of hybrid index and interclass heterozygosity, against which observed combinations can be meaningfully compared.

When considering sites with fixed differences between parental groups, it follows that the hybrid index and interclass heterozygosity of an individual equate to the genome-wide frequency of the p_1_ allele and p_12_ genotype, respectively. Under HWE, genotype frequencies are given by allele frequencies, such that the frequency of the p_12_ genotype across an individual genome is calculated as 2(p_1_)(1 - p_1_), where p_1_ is the frequency of the p_1_ allele. Assuming HWE, if a cross occurs between two individuals with the same p_1_ allele frequency across the genome, the allele frequency will not change in the offspring and the genotype frequency of p_12_ is given by 2(p_1_)(1 - p_1_). Thus, on a triangle plot, the offspring will occur along the curve: p_12_ = 2(p_1_)(1 - p_1_). Alternatively, if a cross occurs between two individuals with different p_1_ allele frequencies, the expected genotype frequencies must be calculated by accounting for the variance in allele frequency between the two parents. This issue is essentially the inverse of the Wahlund effect, which describes the deficiency of heterozygotes in a structured population (Wahlund 1928). Variance is calculated as:

1 $${{\rm{\sigma }}}^{2}=(\sum {({p}_{1i}-\overline{{p}_{1}})}^{2})/{\rm{N}}$$

where p_1*i*_ is the frequency of the p_1_ allele in the i^th^ parent, $\mathop{{p}_{1}}\limits^{\bar{} }$ is the average frequency of the p_1_ allele in the parents, and N is the number of parents (which is always two). The frequency of each offspring genotype is then:

2 $$\begin{array}{c}{\rm{E}}[{{\rm{p}}}_{11}]={(\overline{{p}_{1}})}^{2}-{{\rm{\sigma }}}^{2}\\ {\rm{E}}[{{\rm{p}}}_{12}]=2(\overline{{p}_{1}})\,(1-\overline{{p}_{1}})+2{{\rm{\sigma }}}^{2}\\ {\rm{E}}[{{\rm{p}}}_{22}]={(1-\overline{{p}_{1}})}^{2}-{{\rm{\sigma }}}^{2}\end{array}$$

(Equation (5.1) in Hahn 2018)

We note that in our Eq. (2), the signs for the variance are flipped because we are calculating the expected frequency of each genotype accounting for different allele frequencies, while those in Eq. (5.1) by Hahn (2018) calculate the deviation of expected genotype frequencies from HWE. If both parents have the same p_1_ allele frequency, then the variance is 0 and the calculation of offspring genotype frequencies reduces to the standard formula under HWE. If the parents have different p_1_ allele frequencies, the hybrid index of the offspring will be the same as for a cross of two parents of the average p_1_ allele frequency, because the p_11_ and p_22_ genotypes will decrease by the same increment and changes in the frequency of the p_12_ genotype do not change the hybrid index. For offspring of parents with different p_1_ allele frequencies, the frequency of the p_12_ genotype will always be higher than for a cross of two parents of the average p_1_ allele frequency, because the variance is always positive. Therefore, under HWE, it is impossible for any cross to result in offspring below the curve defined by p_12_ = 2(p_1_)(1 - p_1_), because if the parents have the same allele frequencies their offspring will occur on that curve and if the parents have different allele frequencies their offspring will occur above it. To illustrate this point, we used Eq. (2) to calculate and plot all possible combinations of hybrid index and interclass heterozygosity through six generations, starting with only the genotype frequencies of two parental individuals (Supplementary Fig. S1).

### Simulations

#### Simulation design

To validate our methods for building triangle plots, we simulated genetic data for hybridization between two parental groups at three levels of divergence: low, medium, and high, measured by the number of fixed differences between parental populations. We used SLiM 3 to perform forward-time genetic simulations under a non-Wright-Fisher model (Haller and Messer 2019). Simulations were performed similarly to those in Wiens and Colella (2024), and are briefly summarized here, with details in Supplementary File 1. Each simulation consisted of three phases, with Phase I modeling a common ancestral population prior to divergence. At the start of Phase II, the ancestral population was split into two allopatric populations, which diverged for different numbers of generations (low: 750, medium: 1000, high: 2000 generations). In Phase III, the parental populations (p0, p20) expanded across a gradient of 21 stepping-stone populations to model a contact zone where admixture began in a central region (p10) and introgression occurred into parental populations over time. Once contact was initiated in Phase III, we identified fixed differences between all individuals of the parental populations as a way of tracking true introgression over time. Subsequently, random samples of twenty individuals from each population were taken every 200 generations for 6000 total generations.

To generate known hybrids and parentals, variant sites across all parental genomes were output in VCF format at the end of Phase II. Using the parental genomes, four hybrid classes (F1s, F2s, backcrosses in each direction) were simulated using custom R scripts. Specifically, twenty individuals from each parental population were randomly selected and paired with an individual from the other parental population, and twenty F1s were created by randomly choosing one allele from each parent at each genotype. Twenty F2s were created in the same way, but by pairing each F1 with another F1. Twenty backcrosses in each direction were created by pairing each F1 with a randomly chosen parental individual. Hereafter, we refer to this dataset as “known hybrids and parentals”.

#### Number of individuals sampled from parental populations

The accuracy of observed allele frequencies in each parental population depends on the number of individuals sampled. When an allele occurs at low frequency in a population, the probability of detection increases as the sample size increases. We tested how sample sizes influence the number of AIMs that appear as fixed differences. Using the simulated dataset of known hybrids and parentals, we calculated the difference in allele frequency between parental populations at every variable site. Because all parental individuals were sampled for this dataset, these are the true allele frequency differences between the parental populations. We then randomly downsampled 20, 10, 5, and 2 individuals from each parental population, and used only those samples of individuals to calculate allele frequency differences. We repeated this procedure 200 times for each sample size. We report the distribution of true allele frequency differences at sites that appear to have fixed differences based on each replicated sample size. We then randomly chose one replicate of each sample size with which to calculate hybrid index and interclass heterozygosity of the hybrids and sampled parentals, based on sites with apparent fixed differences (δ = 1).

#### Allele frequency difference thresholds

For the dataset of known hybrids and parentals, we identified AIMs and built triangle plots. We tested three allele frequency difference thresholds for identifying AIMs: δ = 1, δ = 0.75, and δ = 0.5. First, we identified AIMs using all parental individuals in the simulation such that observed allele frequencies in each parental population were the true allele frequencies. Since sampling every parental individual is not feasible for empirical datasets, we also tested δ thresholds using samples of five parental individuals. Using AIMs that passed each allele frequency difference threshold, we calculated hybrid index and interclass heterozygosity for every hybrid and parental individual and built triangle plots.

#### Quantifying error in hybrid index and interclass heterozygosity estimates

We compared *triangulaR* and *bgchm* by calculating mean absolute error (MAE) in hybrid class and interclass heterozygosity estimates for the simulated data containing known hybrid classes. For both methods, we used sets of five parentals and twenty parentals to identify AIMs (δ = 1). We estimated hybrid index and interclass heterozygosity in *triangulaR* for F1s, F2s, backcrosses, and parental individuals. Using the same sets of AIMs, we estimated hybrid index and Q10 in *bgchm* with the default settings of four HMC chains, 2000 steps with no thinning, and 1000 warm-up iterations. We calculated MAE as the difference in observed estimates from the expected values of hybrid index and interclass heterozygosity, based on known hybrid class. MAE in hybrid index and interclass heterozygosity estimates for parental groups were estimated by including twenty individuals of each parental group without assigning them as the parental population used for calling AIMs.

Parental population sample size, δ, and sequencing depth are all expected to contribute to error in hybrid index and interclass heterozygosity estimates. We quantified the effects of each of these variables on error of *triangulaR* estimates by calculating accuracy and precision separately for four hybrid classes (F1, F2, and both backcrosses). We simulated the effect of depth by randomly drawing n alleles from the known genotypes of each simulated individual, where n is the simulated depth. Sites were recoded as heterozygous if at least one of each allele was present in the sample and homozygous if only one allele was present. Accuracy is defined as the difference between the expected value and the average observed value, divided by the expected value. We subtract this value from 1 to report percent accuracy.

3 $${\rm{Accuracy}}=1-(|{\rm{average}}\,{\rm{observed}}-{\rm{expected}}|)/{\rm{expected}}$$

Precision is defined as the average absolute Euclidean distance of each individual estimate from the average estimate.

4 $${\rm{Precision}}=\sum |{\rm{observed}}-{\rm{average}}|/{\rm{N}}$$

Precision is not subtracted from 1 or divided by the expected value, therefore the units reflect the Euclidean distance on the triangle plot, with smaller values indicating higher precision.

#### Introgression and misspecification of parental groups

Identifying AIMs relies on the presence of diagnostic sites across the genomes of the parental groups. To investigate how introgression between parental groups influences AIM identification and calculations of hybrid index, we analyzed generations 0 through 6000 of the simulated data. To track introgression over time, we identified sites with fixed differences in the parental populations (p0 and p20) at generation 0. We refer to these sites as “true” AIMs because they represent real differences prior to gene flow. We then created two additional sets of AIMs for each sampled generation by identifying sites with fixed differences (δ = 1) and sites with allele frequency differences above 0.75 between the samples of the parental populations. In this way, we tested the accuracy of inferred hybrid indices in the face of introgression into the parental populations over time. For each set of AIMs, we calculated the average hybrid index of each population for generations 0 through 6000 from Phase III.

Another assumption of AIM identification is that individuals assigned to the parental groups are sampled from the true parental populations. When sampling natural populations, it is not always known where on the landscape admixture ends and parental populations begin, nor are parentals and hybrids always phenotypically distinguishable. We investigated how misassignment of individuals to parental groups influenced estimates of hybrid index and interclass heterozygosity using generation 1000 of the high divergence simulation as an example where admixture had reached all populations except the parentals. We identified AIMs using five individuals from the parental populations (p0 and p20) and five individuals from partially admixed populations (p5 and p15). Using both sets of AIMs, we then calculated hybrid index and interclass heterozygosity for individuals from every population. We quantified the effect of misassignment of individuals to parental groups compared to using individuals from the correct parental populations by calculating the log-fold change in hybrid index and interclass heterozygosity estimates. To appropriately account for the fact that hybrid index has limits [0,1] and that the parental groups are positioned arbitrarily at either end of this continuum, we subtracted hybrid index estimates greater than 0.5 from 1. This procedure allows log-fold change to represent the nature of the hybrid index interval, in that there are inclusive limits at 0 and 1. Negative values therefore represent shifts towards the nearest parental population when parental populations are misassigned, and positive values represent shifts away from the nearest parental population.

### Empirical example

We tested our method for building triangle plots on a RADseq dataset from a contact zone between two closely related species of songbirds, the sooty and red fox sparrows (*Passerella iliaca* and *P. unalaschcensis*; taxonomy following Gill et al. 2024). These two species come into breeding contact in a narrow region of south-central Alaska (DeCicco 2021). We sequenced 37 specimen-vouchered tissues from a contact zone where parental and intermediate phenotypes occurred together (Table S4, DeCicco 2021). Individuals from allopatric populations of both species (*P. iliaca* *N* = 18, *P. unalaschcensis* *N* = 5) were also sampled and assigned as the parental populations for AIM identification. We identified SNPs following standard bioinformatic and quality filtering workflows documented in DeRaad (2022) and DeRaad et al. (2022, 2023). Plumage patterns were scored following descriptions in DeCicco (2021) to assess correlation between phenotypic and genotypic estimates of hybrid index.

We filtered for 90% and 100% completeness across SNPs. Unless otherwise stated, the 100% complete SNP dataset was used for analysis. To assess influence of uneven sampling of parental populations, we identified AIMs in two ways: (1) using all allopatric *P. iliaca* (*N* = 18) and *P. unalaschcensis* (*N* = 5) as parental populations, or (2) using even parental sampling by downsampling the *P. iliaca* parental population to five individuals. The remaining thirteen *P. iliaca* individuals were retained in the dataset, but not assigned to their parental group. For both sets of parental sampling we identified AIMs (δ = 1) with *triangulaR* and compared estimates of hybrid index and interclass heterozygosity from *triangulaR* to those from *bgchm*. We ran *bgchm* using the default HMC conditions to estimate hybrid index and Q10. Using even parental sampling, we also identified AIMs and built triangle plots with *triangulaR* under three allele frequency difference thresholds (δ = 1, δ = 0.75, and δ = 0.5) for the 90% complete and 100% complete SNP datasets.

## Results

### Summary statistics

The parental populations of the simulated data were sampled at the end of Phase II (allopatric divergence) to create the dataset of known hybrids and parentals. Summary statistics are shown in Table 1. We calculated d_XY_ and π in *pixy*, using variant sites only, to facilitate comparison between the simulated and empirical datasets (Korunes and Samuk 2021). The empirical example of *Passerella* sparrows exhibited similar levels of differentiation and nucleotide diversity as our simulated data (Supplementary Fig. S4, Table 1). When contact began during Phase III of the simulations, there were 28 (low), 59 (medium), and 345 (high) fixed differences between the parental populations. Those fixed differences were used to track introgression into the parental populations during Phase III. The reason for more fixed differences than at the end of Phase II is because it took time for individuals to expand across the gradient and meet in p10, which allowed more fixed differences to accumulate through mutation and drift. Specifically, 241 (low), 269 (medium), and 260 (high) generations elapsed between the end of Phase II and the beginning of contact in Phase III. Although d_XY_ and average δ between parental populations were not substantially different between the low, medium, and high divergence simulations, these levels of divergence provided very few to many fixed differences, which was our main consideration for evaluating the effects of sample size and allele frequency difference threshold on AIM identification.

**Table 1:** Summary statistics for simulated and empirical data.

	Variable sites	Fixed differences	Average δ	d_XY_	π
*Simulated*					
Low	6,251	15	0.10	0.09	p0 = 0.056 p20 = 0.058
Medium	6,511	36	0.11	0.10	p0 = 0.056 p20 = 0.055
High	6,894	297	0.16	0.15	p0 = 0.055 p20 = 0.057
*Empirical*					
*Passerella* sparrows (100%)^a^	13,188	23	0.09	0.11	*ili* = 0.102 *una* = 0.062

All statistics for the simulated data were calculated using all parental individuals (p0 and p20) at the end of Phase II (allopatric divergence).

^a^Statistics calculated using the dataset with 100% completeness across SNPs and all allopatric *P. iliaca* (*ili*; *N* = 18) and *P. unalaschcensis* (*una*; *N* = 5) samples.

### Quantifying error in hybrid index and interclass heterozygosity estimates

**Fig. 2:**
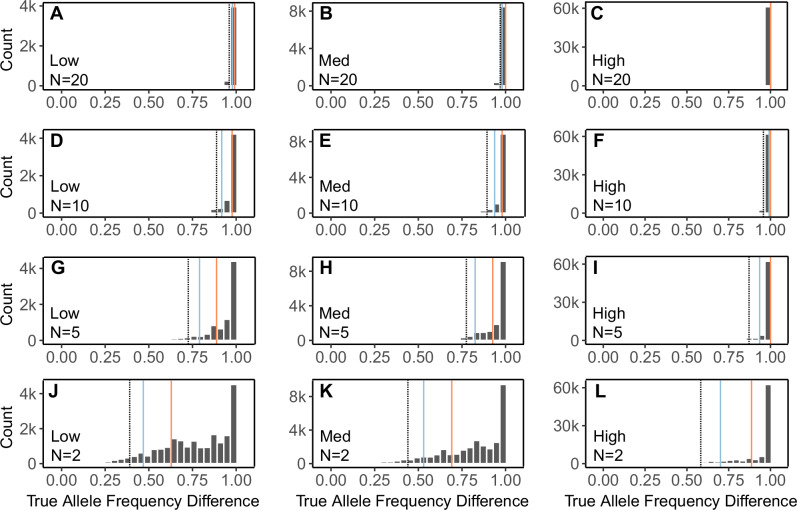
True allele frequency differences of sites with fixed differences in the parental samples. Distributions, based on simulated data, of the true allele frequency differences of sites that appear to have fixed differences with smaller parental sample sizes: 20 (**A**–**C**), 10 (**D**–**F**), 5 (**G**–**I**), and 2 (**J**–**L**). The observed distributions were created with 200 random sampling replicates of 20, 10, 5, or 2 individuals (N) from each parental population. The left column shows the simulation with low differentiation, center shows the simulation with medium differentiation, and the right column shows the simulation with high differentiation. On each plot, 95% of observed values fall above the black dotted line, 90% of the values fall above the blue solid line, and 75% fall above the red solid line.

The accuracy of estimated allele frequency differences increased as the sample size of the parental populations increased (Supplementary Table S2). With a sample of twenty individuals per parental population the average accuracy of estimated allele frequency differences was >99% for all levels of divergence. A sample size of five per parental population also yielded high accuracy, at 93%, 95%, and 98% for low, medium, and high levels of divergence, respectively. The distribution of true allele frequency differences at sites with fixed differences in the sample are consistently left-skewed, with a peak at one (Fig. 2). With a sample size of five per parental population, 95% of sites with δ = 1 in the sample had a true allele frequency difference of at least 0.72 for all levels of divergence.

**Fig. 3:**
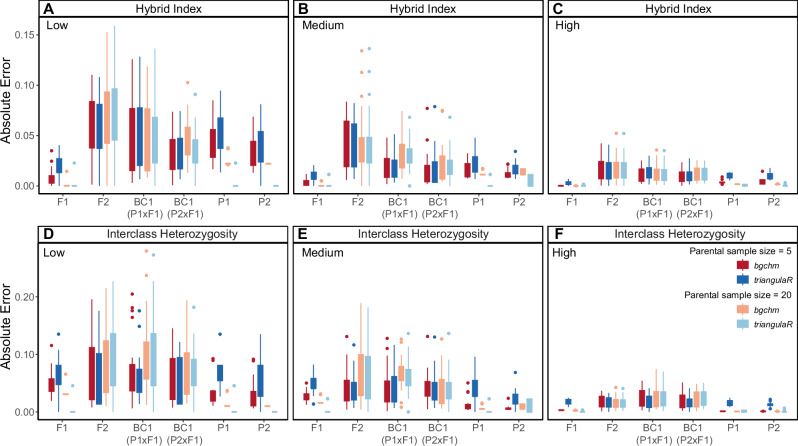
Error estimates for *triangulaR* and *bgchm*. Absolute error in hybrid index (**A**–**C**) and interclass heterozygosity (**D**–**F**) estimates for known simulated hybrid classes using *triangulaR* and *bgchm*. AIMs (δ = 1) were identified using two different parental sample sizes (*N* = 5, *N* = 20). The low divergence simulation is shown in the left column, medium divergence in the middle, and high divergence in the right. Absolute error for each method is summarized for each hybrid class by a box plot.

We compared MAE for *triangulaR* and *bgchm* for parental sample sizes of five and twenty, finding little difference in MAE between the programs (Fig. 3). For both programs, MAE was generally highest when using five samples from each parental population, and decreased with an increased sample size of twenty. Independent of parental population sample size, MAE decreased for all hybrid classes as divergence between the parental populations increased.

**Fig. 4:**
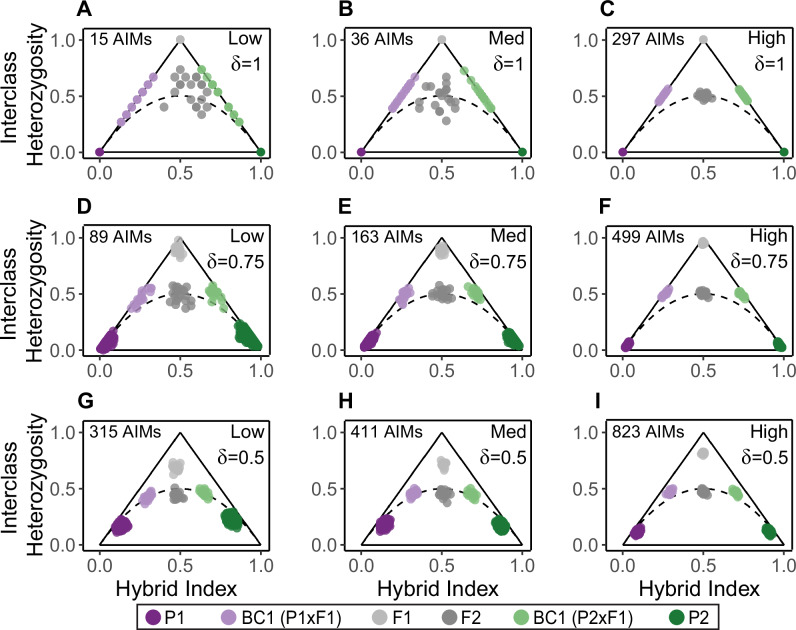
Triangle plots built with AIMs under different allele frequency difference thresholds. Triangle plots for known hybrids and parentals based on AIMs identified with true allele frequency difference thresholds (δ) of 1 (**A**–**C**), 0.75 (**D**–**F**), and 0.5 (**G**–**I**). The left column shows the simulation with low differentiation, the center column shows the simulation with medium differentiation, and the right column shows the simulation with high differentiation. Solid black lines indicate the possible space on a triangle plot, and the dotted black curve indicates the boundary below which individuals cannot occur, assuming Hardy-Weinberg Equilibrium.

**Fig. 5:**
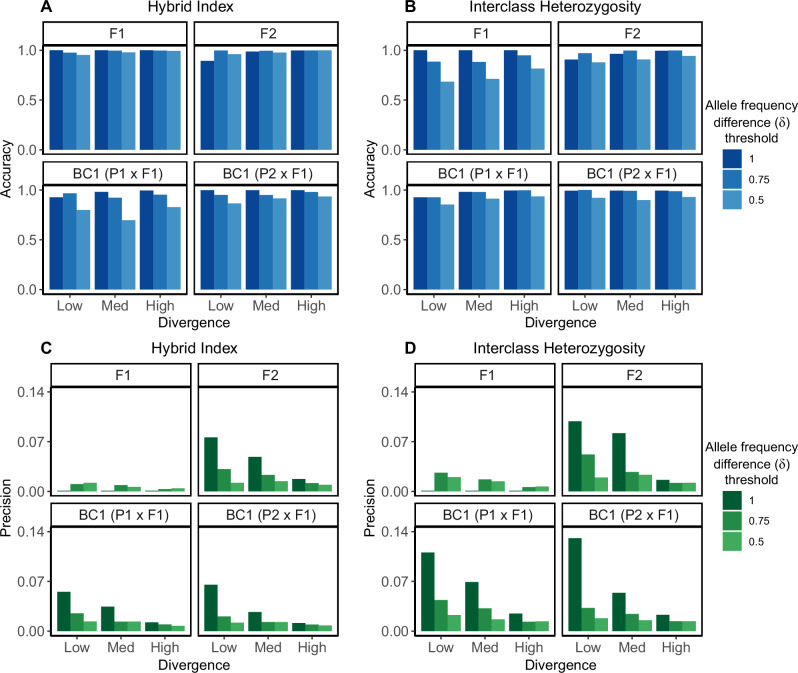
Accuracy and precision of hybrid index and interclass heterozygosity estimates in triangulaR. Accuracy (**A**, **B**) and precision (**C**, **D**) of hybrid index (**A**, **C**) and interclass heterozygosity (**B**, **D**) estimates based on AIMs identified with true allele frequency difference thresholds of 1, 0.75, and 0.5. Each simulation (low, medium, high) is shown on the x-axis. Accuracy and precision was measured for 20 individuals from each of the four hybrid classes (F1, F2, and the two first generation backcrosses) separately. Accuracy is reported as a percent, with 1 indicating 100% accuracy. Precision is reported as the average Euclidean distance of each observation within a class from the average of that class, such that smaller values indicate higher precision.

Estimated values of hybrid index and interclass heterozygosity for hybrids were generally most accurate when δ = 1, except for F2 estimates, which were more accurate when δ = 0.75 (Figs. 4 and 5). For all values of δ, estimates were generally very precise (<0.07 average deviation), with the exception of interclass heterozygosity estimates for F2s and backcrosses when using δ = 1. For all hybrid classes except F1s, estimates became more precise as δ decreased. Estimated values of hybrid index and interclass heterozygosity for each hybrid class remained highly accurate (>90%) down to a sample size of five individuals from each parental population (Supplementary Fig. S6). Estimated values of hybrid index and interclass heterozygosity were also highly precise (<0.1 average deviation) for all sample sizes and levels of divergence. With a sample size of five from each parental population, hybrids appear as expected on triangle plots regardless of the level of divergence between parental populations (Supplementary Fig. S5). Hybrid index estimates were unaffected by depth, remaining highly accurate (97.9% accuracy on average) for simulated depths as low as 2X, assuming no sequencing error (Supplementary Fig. S7). In contrast, interclass heterozygosity estimates were dependent on depth, and only attained high accuracy (>95%) with 6X depth or higher.

### Empirical example

**Fig. 6:**
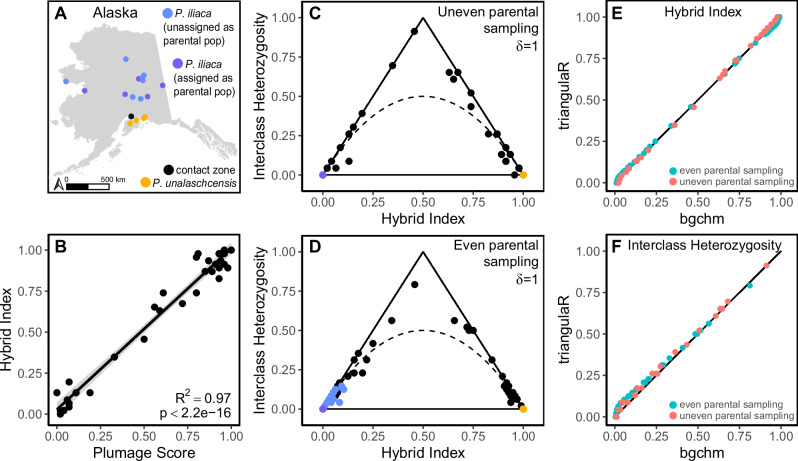
Estimations of hybrid index and interclass heterozygosity for an empirical example. **A** The location of fox sparrow (*Passerella* spp.) specimens used in this example. **B** Linear regression between plumage score and genetic hybrid index for *Passerella* specimens from the contact zone. All allopatric individuals (18 *P. iliaca*, 5 *P. unalaschcensis*) were assigned to the parental populations to identify AIMs (δ = 1), and hybrid index was calculated in *triangulaR*. **C** Triangle plots built in *triangulaR* using all allopatric individuals assigned as parentals (uneven parental sampling) and **D** five allopatric individuals assigned as parentals (even parental sampling) to identify AIMs (δ = 1). Estimates of **E** hybrid index and **F** interclass heterozygosity from *triangulaR* were compared to those from *bgchm*, using AIMs (δ = 1) from both sampling schemes.

The 90% and 100% completeness filters for the empirical example (*Passerella* spp.) retained 24,592 and 13,188 SNPs, respectively. Using AIMs identified with δ = 1, hybrid index estimates aligned with expectations based on plumage scores (Fig. 6A, B). There were some minor differences in hybrid index and interclass heterozygosity estimates when all available allopatric *P. iliaca* (*N* = 18) were assigned as the parental group compared to the downsampled dataset in which five allopatric *P. iliaca* were assigned as the parental group (Fig. 6C, D). Most notably, the thirteen allopatric *P. iliaca* that were not assigned as the parental group had increased hybrid index and interclass heterozygosity estimates in relation to when they were assigned as the parental group, overlapping with some individuals from the contact zone. That pattern suggests the presence of parental *P. iliaca* individuals in the contact zone. All estimates made using *triangulaR* were highly comparable to those of *bgchm* (Fig. 6E, F).

### Introgression and misspecification of parental populations

In each simulation, some alleles at sites that had fixed differences at the beginning of contact had introgressed into both parental populations (p0 and p20) by generation 2000 (Supplementary Fig. S8). By generation 6000, each parental population contained at least 25% ancestry from the other parental population at sites that had started as fixed differences. Yet, the estimated ancestry of each parental population using AIMs (δ = 1) identified with twenty sampled parentals remained at 0 and 1 for p0 and p20, respectively, across all generations. Lowering δ to 0.75 resulted in the recognition of some admixture in the parental populations, but did not recover true levels of introgression into the parental populations.

When individuals that have experienced some admixture were misassigned to the parental groups, the largest fold-change in hybrid index and interclass heterozygosity estimates occurred in individuals with small proportions of admixture (Supplementary Fig. S9). In our simulation, hybrid index and interclass heterozygosity estimates for individuals from the same population (p5 and p15) as the individuals misassigned as parentals became more similar to their nearest true parental population (p0 or p20), indicated by negative log-fold changes. Individuals from the true parental populations experienced large shifts away from their true hybrid index and interclass heterozygosity values, in some cases with estimates quadrupling the true value. Individuals with intermediate admixture proportions experienced less dramatic shifts in hybrid index and interclass heterozygosity estimates. Average log-fold changes in hybrid index and interclass heterozygosity for individuals with true hybrid index values between 0.3 and 0.7 were −0.07 and −0.02, respectively.

## Discussion

Here, we present *triangulaR* (https://github.com/omys-omics/triangulaR), an R package for identifying AIMs, calculating hybrid index and interclass heterozygosity, and visualizing triangle plots from SNP data. To facilitate use of *triangulaR* and interpretation of results, we simulated data to examine how two common criteria for identifying AIMs, the sample size of parental groups and the allele frequency difference threshold, influence estimation of hybrid index and interclass heterozygosity. Further, we provide a set of expectations for the covariance of hybrid index and interclass heterozygosity under HWE, against which empirical data can be compared. We anticipate *triangulaR* and the theoretical framework developed here will be useful for identifying hybrids, assigning individuals to hybrid classes, designing sampling schemes for natural populations, analyzing next-generation sequencing data, and interpreting triangle plots.

### Recommendations for study design and interpretation of triangle plots

#### How to choose an allele frequency difference threshold for AIM identification

There is a tradeoff between setting a high allele frequency difference threshold, which will recover fewer, more informative sites, versus selecting a lower threshold that will recover more, potentially less informative sites. The optimal threshold will depend on the study system, but as a best practice various thresholds should be explored during the analysis of empirical data. Our simulations suggest that the most important factor for accuracy and precision of hybrid index and interclass heterozygosity estimates is the number of AIMs used for calculations (Fig. 5 and Supplementary Fig. S6). This is especially true when the level of divergence between parental groups is low. For example, using only the 15 fixed differences present between the parental populations in the low divergence simulation, we obtained perfect estimates of hybrid index and interclass heterozygosity for parentals and F1s, but imprecise estimates for F2s and backcrosses. When δ is relaxed to 0.75, almost six times as many AIMs (*N* = 89) are recovered, and the precision and accuracy of hybrid index and interclass heterozygosity estimates increased for F2s and backcrosses, with minimal decrease in accuracy or precision of the estimates for F1s. F2 and backcross estimates are more sensitive to the number of AIMs used because there are three (p_11_, p_12_, p_22_) and two (p_11_, p_12_) possible genotypes at each AIM in these genomes, respectively, while there is only one genotype (p_12_) expected at each AIM in F1 genomes.

Additional patterns emerge when comparing triangle plots built for the same individuals but with AIMs called at different δ thresholds (Fig. 4 and Supplementary Fig. S12). For F1s, the accuracy and precision of interclass heterozygosity estimates decrease as the δ threshold decreases, accompanied by a downward shift in the estimated values themselves, while the accuracy and precision of hybrid index estimates are largely unaffected. For backcrosses, interclass heterozygosity estimates remain accurate and become more precise as the δ threshold decreases, but estimated values of hybrid index shift towards the center of the plot (0.5). Taken together, a trend emerges wherein all hybrid classes gravitate towards the center of the triangle plot at lower δ thresholds. This pattern becomes less pronounced when divergence between parental groups is higher, but should be taken into consideration when qualitatively inferring hybrid classes based on a triangle plot.

When analyzing empirical data, it is best practice to try multiple allele frequency difference thresholds and compare resulting triangle plots. If many (>100) AIMs are recovered with δ = 1 and the resulting triangle plots are mostly unchanged with lower values of δ, then choosing δ = 1 is likely appropriate. Even as few as 30 fixed differences can produce reliable triangle plots, but selecting a lower threshold (e.g. δ = 0.75) may produce more precise estimates without sacrificing accuracy. When there are very few (<15) to no fixed differences between the parental populations, lower δ thresholds that provide more AIMs will be necessary to distinguish hybrid classes. We emphasize, however, that the reason lower δ thresholds can provide better estimates is because they increase the total number of AIMs. If few (<50) AIMs are identified even with a low threshold (e.g. δ = 0.5), accurate and precise hybrid index and interclass heterozygosity estimates will be difficult or impossible to obtain (Supplementary Figs. S10 and S11).

#### What sample size of parental groups is needed?

To identify AIMs with which to calculate hybrid index and interclass heterozygosity, samples from both parental groups are required. The goal is to sample individuals that have not experienced admixture, such that they accurately reflect allele frequencies in the respective parental populations. Thus, an important practical consideration in experimental design is the number of parental individuals needed to provide reasonably accurate estimation of allele frequency differences between populations.

We describe the distribution of true population-wide allele frequency differences of AIMs that falsely appear as fixed differences in the parental group samples. When divergence between the parental groups is low, it is difficult to minimize the proportion of false positive fixed differences. In our simulation with the least divergence between parental populations (true fixed differences at only 0.2% of variable sites), a sample size of twenty individuals per parental population and δ = 1 resulted in a 29% false positive rate. That means that of the sites that appeared to be fixed for different alleles in the sample, 29% were not actually fixed in the population. Yet, it is important to consider the distribution of true allele frequencies of those perceived fixed differences. Sites with a high allele frequency difference between the parentals still provide valuable information, even if they are not fixed. With a sample size of twenty individuals from each parental population, >95% of identified AIMs (δ = 1) had a true allele frequency difference >0.95 for all simulated levels of divergence (Fig. 2, Supplementary Table S2). Across all sample sizes, the distribution of true allele frequency differences at sites that appear to be fixed differences based on the sample is always left-skewed, meaning that the noise caused by the inclusion of some sites with low δ is minimal.

Counterintuitively, our simulations suggest that a sample size of five from each parental population can provide more precise estimates of hybrid index and interclass heterozygosity than larger sample sizes, without sacrificing accuracy (Supplementary Fig. S6). That is because more sites pass the δ = 1 threshold when the parental sample size is smaller, resulting in a larger set of AIMs. While some of those sites are false positives in the sense that they are not truly fixed differences, they still exhibit large allele frequency differences between the parental populations (Fig. 2). As such, precision is increased not because of the lower sample size per se, but through the inclusion of additional informative sites obtained with the lower allele frequency difference threshold.

In empirical systems, the optimal number of parental individuals to sample will depend on the question, level of divergence, and sequencing effort, among other variables. For deeply diverged species, hybrid index and interclass heterozygosity can be accurately estimated with as few as two or three samples from each parental species, whereas more shallowly diverged groups may require a parental sample size of twenty or more. But as a general rule of thumb, a sample of five individuals from each parental population will usually be sufficient to address questions related to hybridization, provided there is enough sequencing effort to obtain thousands of SNPs. We also note that in our simulations, decreasing the parental sample size from twenty to five only marginally affected the accuracy of hybrid index and interclass heterozygosity estimates, and precision actually increased for F2s and backcrosses. Hybrid classes also appeared as expected on triangle plots with parental sample sizes as few as five individuals (Supplementary Fig. S5), an important consideration, as hybrid classes are often inferred qualitatively based on the combination of hybrid index and interclass heterozygosity (Fitzpatrick 2012).

#### Interpreting triangle plots

Triangle plots are not only useful for identifying hybrids and assigning hybrid classes, but have also been used as support for the presence/absence of barriers to reproduction (Fitzpatrick 2012; Lindtke et al. 2012; Christe et al. 2016; Pulido-Santacruz et al. 2018). To help guide such inferences, we outline a theoretical framework for triangle plot interpretation based on the expectations of the covariance of hybrid index and interclass heterozygosity under HWE. We demonstrate mathematically that under HWE, the possible space on a triangle plot is defined by the lines y = 2x and y = −2x + 2, and the curve y = 2x(1 − x) (Fig. 1). Deviation from those expectations – most notably, individuals falling below the curve – is consistent with violation of HWE and can therefore provide support for the presence of barriers to reproduction, nonrandom mating, natural selection, and/or genetic drift (Pulido-Santacruz et al. 2018). For example, drift in an admixed population could cause individuals to occur below the curve defined by HWE through the random fixation of alleles over time. If alleles from either parental group have an equal chance of reaching fixation in the hybrid population, then interclass heterozygosity would decrease, but hybrid index would remain unchanged. Importantly, the absence of individuals below the curve defined by HWE is not evidence for barriers to reproduction or natural selection for or against hybrids; on the contrary, such an absence is expected when there is random mating and no selection.

The presence/absence of certain hybrid classes can shed light on hybrid zone dynamics. For example, support for post-zygotic isolation due to Dobzhansky-Muller incompatibilities (DMIs) may be gained due to the absence of F2s (or any individuals with hybrid index and interclass heterozygosity of 0.5), because DMIs are expected to manifest in the F2 generation, when there is no longer at least one copy of each parental allele at every locus (Maheshwari and Barbash 2011; Thompson et al. 2023). This pattern would be reflected on triangle plots by the absence of F2s and the presence of hybrid classes along the outer edges of the triangle. Our empirical example is suggestive of DMIs, because only F1s, backcrosses, and parentals are observed (Fig. 6), although more work and a larger sample is needed to fully support the presence of DMIs in this system.

Consideration of the expectations for triangle plots under HWE also informs inference of hybrid classes. F1s and first generation backcrosses are easily distinguished because their combination of hybrid index and interclass heterozygosity is unique. F2s, however, are indistinguishable from F3s and further crosses, because under HWE all are expected to have a hybrid index and interclass heterozygosity of 0.5. We also show that after only four generations of unidirectional backcrosses to one parental group, hybrid index (0.03125) and interclass heterozygosity (0.06250) will be only marginally different from members of the backcrossing parental group, making distinctions based on these metrics difficult. Further, we show through simulations and an empirical example that it is difficult to minimize error for estimates of hybrid index and interclass heterozygosity for known parentals when they are not assigned as the parental group used for calling AIMs. As a result, the estimates for unassigned parentals can easily be in the range of those for late generation backcrosses (Supplementary Fig. S9). For example, in our fox sparrow dataset, the thirteen allopatric *P. iliaca* that are not assigned as the parental group have estimated interclass heterozygosity values on par with those of individuals from the contact zone (Fig. 6). It is therefore difficult to confidently assign those contact zone individuals as highly backcrossed or as parentals. We expect, however, that at least some of the sampled individuals from the contact zone are parentals, because we observe an F1 and first generation backcrosses in the contact zone, which must be the offspring of at least one parental individual.

### Some common pitfalls and ways to avoid them

Our method performs well even when there is low divergence between parental groups, provided that there is enough sequencing effort across the genome to identify >30 SNPs with high allele frequency differences. We expect that in most cases, modern sequencing methods (e.g. RADseq, target capture) will provide large enough SNP datasets for this endeavor. However, if divergence between parental groups is extremely low, such that there are no fixed differences and <50 SNPs pass an allele frequency different threshold of 0.5, calculating hybrid index and interclass heterozygosity becomes inaccurate and imprecise. To overcome such limitations, a larger proportion of the genome will need to be sequenced. Sequencing more parental individuals and/or sequencing the same loci at higher coverage will filter out less-informative AIMs, but will not increase the number of AIMs identified, which is the primary driver of accuracy and precision of hybrid index and interclass heterozygosity estimates.

In some cases, Bayesian inference of hybrid index and interpopulation ancestry (Q10) could overcome the limitations imposed by datasets with few diagnostic AIMs (Gompert et al. 2024). Implemented in *bgchm*, this approach makes use of sampled parental allele frequencies to estimate the likelihood of hybrid index and Q10 across AIMs, regardless of allelic state. In other words, this approach takes into account the possibility that individuals with a homozygous genotype inherited each allele from a different population. While more computationally intensive than *triangulaR* (Supplementary Table S3), this method has been shown to give accurate estimates for AIMs with δ as low as 0.5 and with parental sample sizes of 50 (Gompert et al. 2024). Whether this method is robust to smaller parental sample sizes and lower divergence between parental groups is not yet clear.

When interpreting triangle plots, it is important to consider the assumptions made when choosing a δ threshold and assigning individuals to parental groups. When using only fixed differences (δ = 1) as AIMs, all individuals assigned as parentals will, by definition, have an interclass heterozygosity of 0 and hybrid index of either 0 or 1. An individual misassigned to either parental group will, therefore, be impossible to diagnose in this context. Thus, even if there are enough fixed differences to justify a threshold of δ = 1, it is still worthwhile to explore lower thresholds, with which it may be possible to distinguish misassigned parentals from true parentals. This approach is most likely to work if divergence between the parental groups is high and there are large sample sizes from each, such that estimates for true parental individuals remain unchanged but those for misassigned individuals do change.

If the parental populations are misspecified entirely, for example by choosing populations that have experienced small degrees of admixture, it can be difficult to detect based on a triangle plot alone. Our simulations show that when partially admixed individuals are assigned as the parental populations, estimates of hybrid index and interclass heterozygosity for nearby individuals are skewed towards those of the true parental populations. For example, when five individuals from our simulated p5 and p15 populations were misassigned as parentals, the estimates for the majority of the remaining 15 individuals from those populations were skewed towards parental values (Supplementary Fig. S9). Even if true parentals are included, the misspecification may not be apparent, because their interclass heterozygosity estimates based on AIMs from a misspecified parental population could be more than double their true value (Supplementary Fig. S9). If true parentals are included, then misspecified parental individuals could be detected using unsupervised clustering approaches such as STRUCTURE (Pritchard et al. 2000) or ADMIXTURE (Alexander et al. 2009), although care should be taken to avoid common misinterpretations of results from those approaches (Bradburd et al. 2018; Lawson et al. 2018; Wiens and Colella 2024).

More problematic is if there are no true parentals in the sample. In the absence of diagnostic morphological features, it may not be possible to detect introgression into parental populations. In our simulations, introgression occurs into the parental populations by generation 2,000, but it is impossible to detect using allele frequency data from that generation on, because sites that began as fixed differences between the two parental groups now occur in both groups. As such, sites that were once informative of ancestry become indistinguishable from shared ancestral variation (Fig. S8). The true history of introgression is therefore obscured by the unknowability of ancestral allele frequencies. Admixture proportions inferred by unsupervised clustering algorithms also face this problem, because the models cannot distinguish admixture in every individual from shared ancestral variation, and subsequently interpret the least-admixed individual(s) to belong entirely to one genetic cluster (Lawson et al. 2018).

### Future directions

Error is introduced into estimates of hybrid index and interclass heterozygosity when the observed allele frequency differences of AIMs between sampled populations differ from the true allele frequency differences. Quantifying this error is exceptionally difficult, because there is no closed-form analytical method for deriving the probability distribution of population allele frequencies based on sampled allele frequencies (Tataru et al. 2017). Bootstrapping across parental samples and/or allele frequencies is ineffective for quantifying error because these methods incorrectly assume the sampled allele frequency is the population allele frequency and because analytical estimates of hybrid index and interclass heterozygosity do not distinguish between identity-by-state and identity-by-descent. Methods for estimating the probability distribution of true allele frequencies include Markov chains and diffusion approximation (Tataru et al. 2017), both of which are computationally intensive, but which could be incorporated into future versions of *triangulaR* to provide error estimates and to identify the most likely hybrid class of each sample.

Other challenges include cases where parental groups are minimally divergent or have experienced introgression. In the case of introgression into parental populations, analyzing the distribution of coalescent heights across the genome may prove effective for distinguishing introgressed loci from shared ancestral variation (Hibbins and Hahn 2022). Additional work is also needed to develop the theoretical expectations for observed combinations of hybrid index and interclass heterozygosity in the presence of pre- and/or post-zygotic reproductive isolation. We expect that *triangulaR* will be a useful community resource for identifying and describing hybridization using genomic data, and that with continued theoretical development, triangle plots will serve as an effective tool for understanding the evolutionary processes governing hybrid zone dynamics.

## Supplementary information

Supplementary_File_1

## Data Availability

The R package *triangulaR* is available from http://github.com/omys-omics/triangulaR. SLiM, Bash, Python, and R scripts used to simulate genetic data and perform analyses are available on GitHub at http://github.com/omys-omics/triangle_plot_sims. Raw VCF output from SLiM simulations are available at the same link. Raw reads for the empirical example are deposited in the NCBI SRA (BioProject PRJNA1243450).
