# Supplementary material for: triangulaR: an R package for identifying AIMs and building triangle plots using SNP data from hybrid zones
Source: Heredity (Edinb). 2025 Apr 12;134(5):251–62. doi: 10.1038/s41437-025-00760-2 (PMC12056084; doi:10.1038/s41437-025-00760-2)
Supplement: Supplementary file 1 — Supplementary_File_1 [file 41437_2025_760_MOESM1_ESM.docx]

**SUPPLEMENTAL INFORMATION FOR:**

***triangulaR:* an R package for identifying AIMs and building triangle plots using SNP data from hybrid zones**

Authors: Ben J. Wiens^1^*, Lucas H. DeCicco^1^, & Jocelyn P. Colella^1^

1. Biodiversity Institute, Department of Ecology and Evolutionary Biology, University of Kansas, Lawrence KS 66045

*Corresponding author: bjwiens@ku.edu

*Simulations*

Each simulation consisted of three phases (Fig. S2). Phase I lasted 1,000 generations and modeled a single, common ancestral population prior to divergence. Two populations existed during Phase I, with a high migration rate between them, such that in each generation 20% of individuals on average switched populations. Migration ceased during Phase II, which simulated allopatric divergence between the two parental populations. The length of Phase II differed for each simulation (low: 750, medium: 1000, high: 2000 generations), in order to reach different degrees of differentiation. Phase III modeled range expansion under a linear, stepping-stone model of 21 populations and lasted for 6,000 generations after contact was initiated between the two parental populations. The two parental populations were situated at the far ends of the simulation “landscape”, and migration could only occur between neighboring populations. Each generation, five migrants from each population with >200 individuals were randomly chosen to move into each adjacent population.

A 2 Mb diploid genome was simulated for each individual. Mutation rate was set to 10^-7^ per site per generation, all mutations were selectively neutral, and the recombination rate was 10^-5^ per site per generation. Those genomic parameters were chosen to generate SNP datasets on the order of magnitude (e.g. thousands) typically obtained from reduced-representation sequencing data, and such that linkage disequilibrium was minimal. Individuals were hermaphroditic, and could only reproduce if they existed in the previous generation. Each generation, every eligible individual reproduced with another randomly chosen individual in the same population. The number of offspring per mating followed a Poisson distribution (λ = 1.04). After all eligible individuals reproduced, they were removed from the simulation, and then migrants were randomly selected from among the offspring and moved to an adjacent population. Because individuals only existed for two generations and the carrying capacity per population was 1,100, the maximum number of eligible migrants per population fluctuated around 550.

At the end of Phase II, variant sites across all parental genomes were output in VCF format, and four hybrid classes (F1s, F2s, backcrosses in each direction) were simulated using custom R scripts. Specifically, 20 individuals from each parental population were randomly selected and paired with an individual from the other parental population, and 20 F1s were created by randomly choosing one allele from each parent at each genotype. Twenty F2s were created in the same way, but by pairing each F1 with another F1. Twenty backcrosses in each direction were created by pairing each F1 with a randomly chosen parental individual. From here on, we refer to this dataset as “known hybrids and parentals”.

During Phase III, random samples of 20 individuals from each population were taken every 200 generations for 6,000 total generations. Variant sites within the sampled individuals from each sampled generation were output in VCF format. The first sample was not taken until contact between the parental populations occurred. To ensure that contact happened in the central-most population (p10), migration could only occur into p10 once both adjacent populations (p9 and p11) contained more than 200 individuals. Once both p9 and p11 contained more than 200 individuals, both populations began sending migrants into p10 simultaneously. We took the first sample once p10 had at least 50 individuals, and define this as the first generation of contact. In this way, the first sample always occurred within the first few generations of hybridization in p10. We refer to that sample as generation 0, and subsequent samples as the number of generations since the first sample. At generation 0, we recorded the sites that contained fixed differences between all individuals of the parental populations, as a way of tracking the true degree of introgression over time


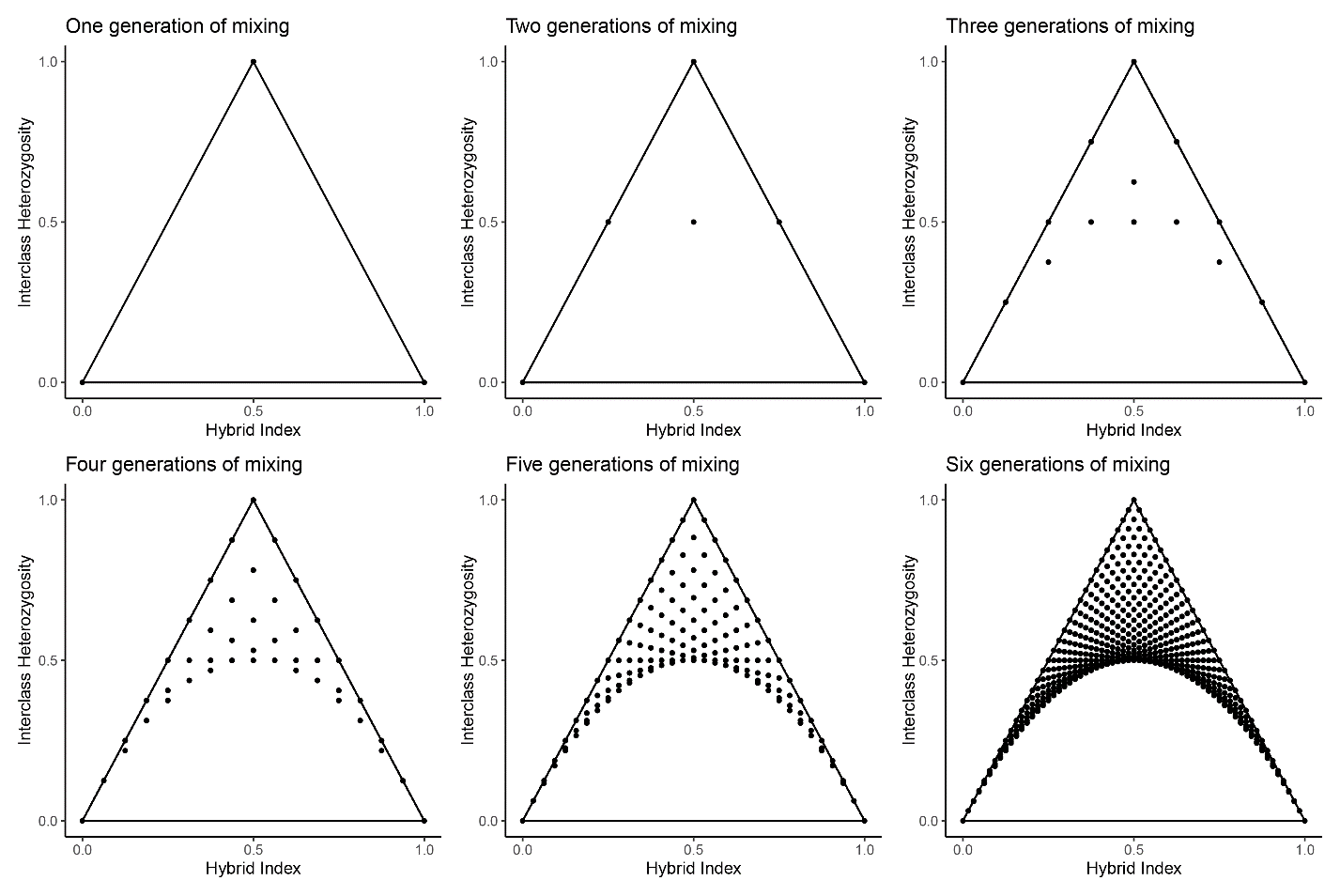


**Figure S1.** Calculation of possible coordinates of individuals on a triangle plot after one, and up to six, generations of all possible matings of all individuals in the previous generation, under Hardy-Weinberg Equilibrium.

**
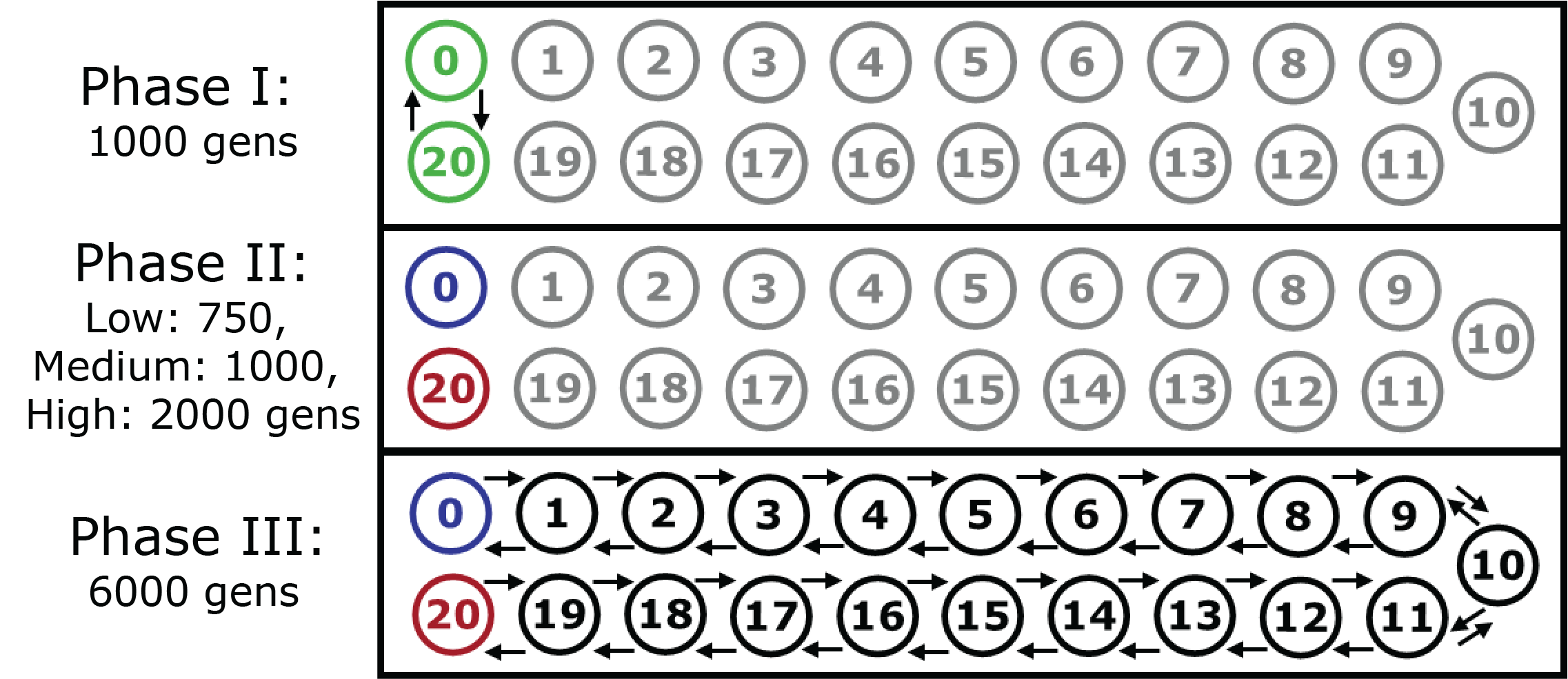
**

**Figure S2.** Schematic of the three phases of the simulations. In Phase I, individuals only occur in the green populations (p0 and p20), which are connected with a high migration rate, and no individuals exist in the gray populations. In Phase II, the parental populations (p0: blue; and p20: red) become allopatric, and evolve independently for 750, 1000, or 2000 generations. In Phase III, individuals expand from the parental populations until contact is made in the central population (pop 10). Migration between each population proceeds under a stepping-stone model.

**
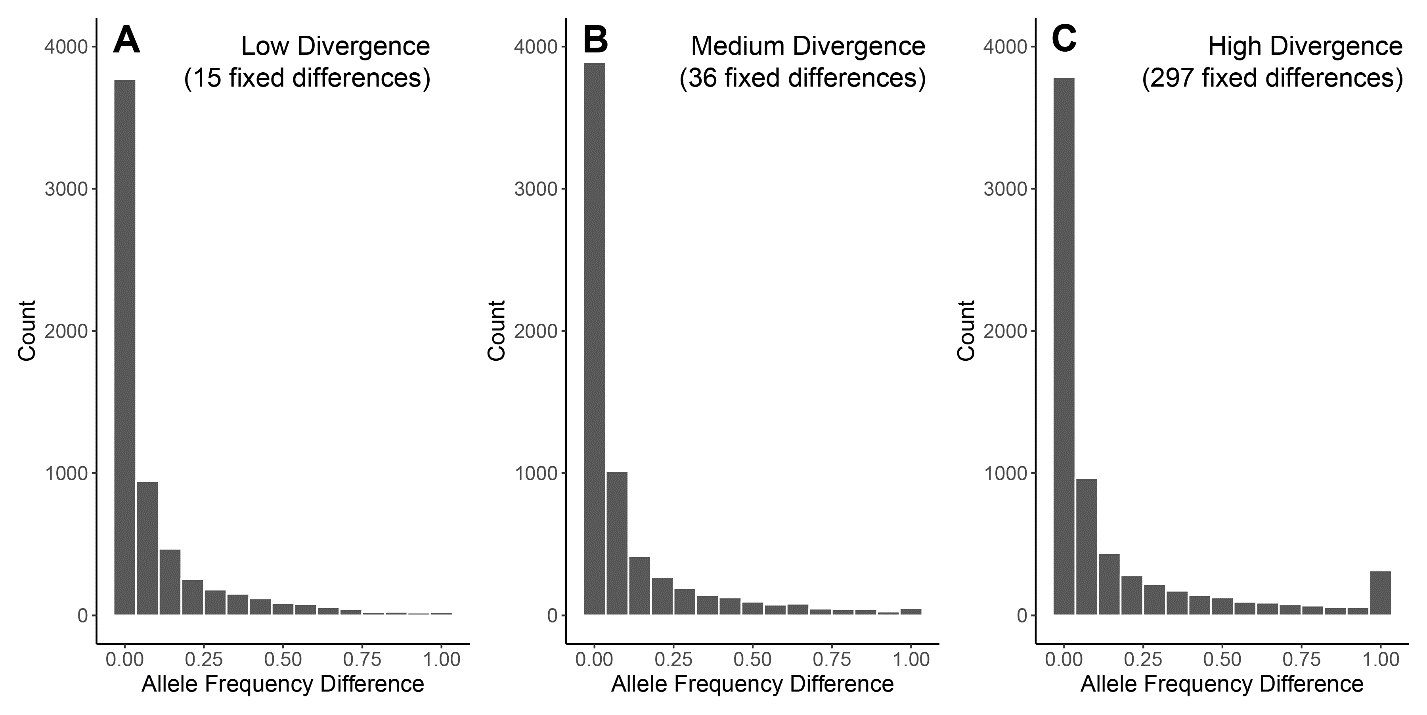
**

**Figure S3.** Spectrum of allele frequency differences between the parental populations in each simulation at the end of Phase II. There were 6251, 6511, and 6894 SNPs in the low **(A)**, medium **(B)**, and high **(C)** divergence simulations, respectively.


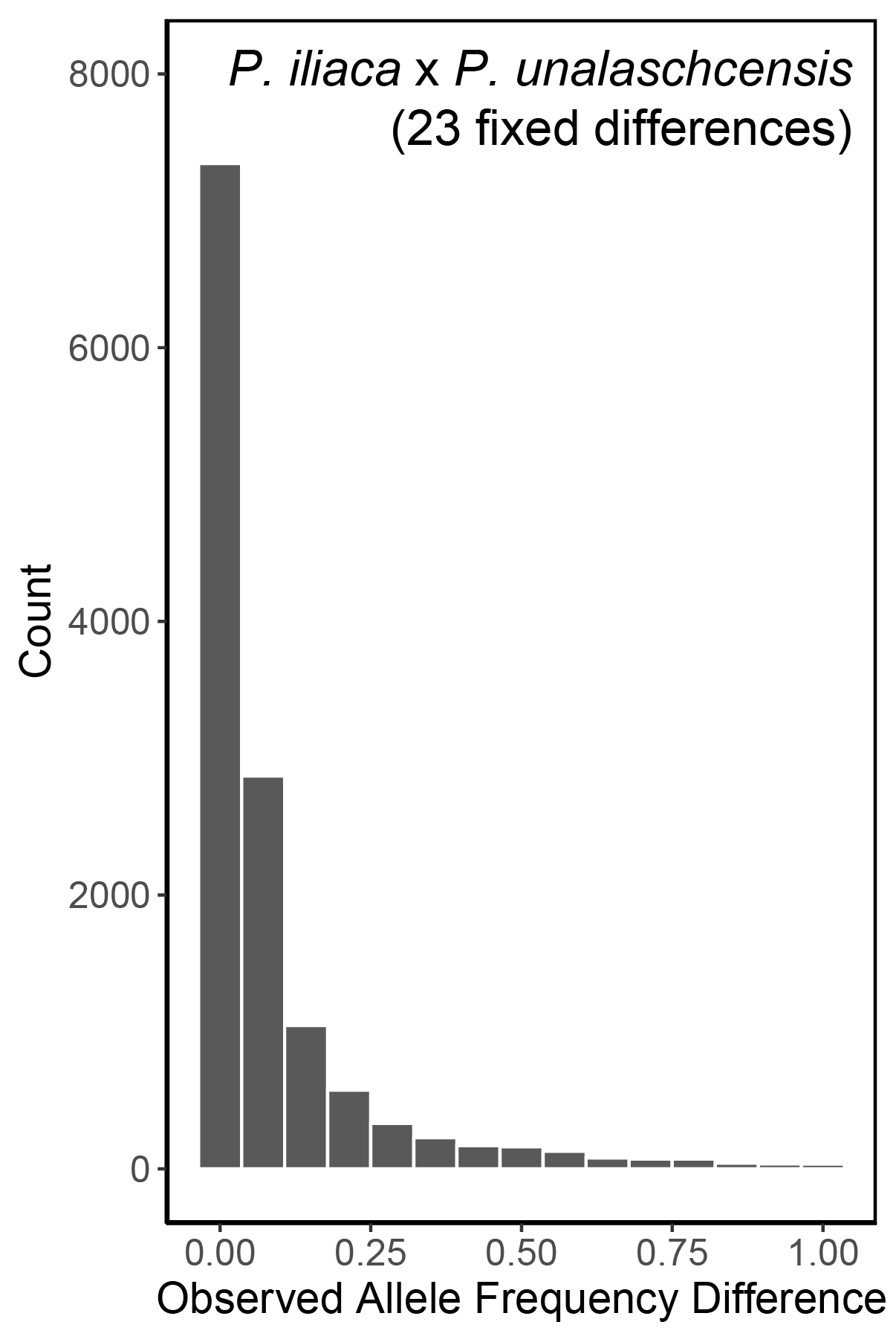


**Figure S4.** Spectrum of allele frequency differences between the parental populations (*P. iliaca*, *P. unalaschcensis* in the empirical dataset. Allele frequency differences were calculated using 18 allopatric *P. iliaca* and 5 allopatric *P. unalaschcensis*, with 100% completeness across 13,188 SNPs.


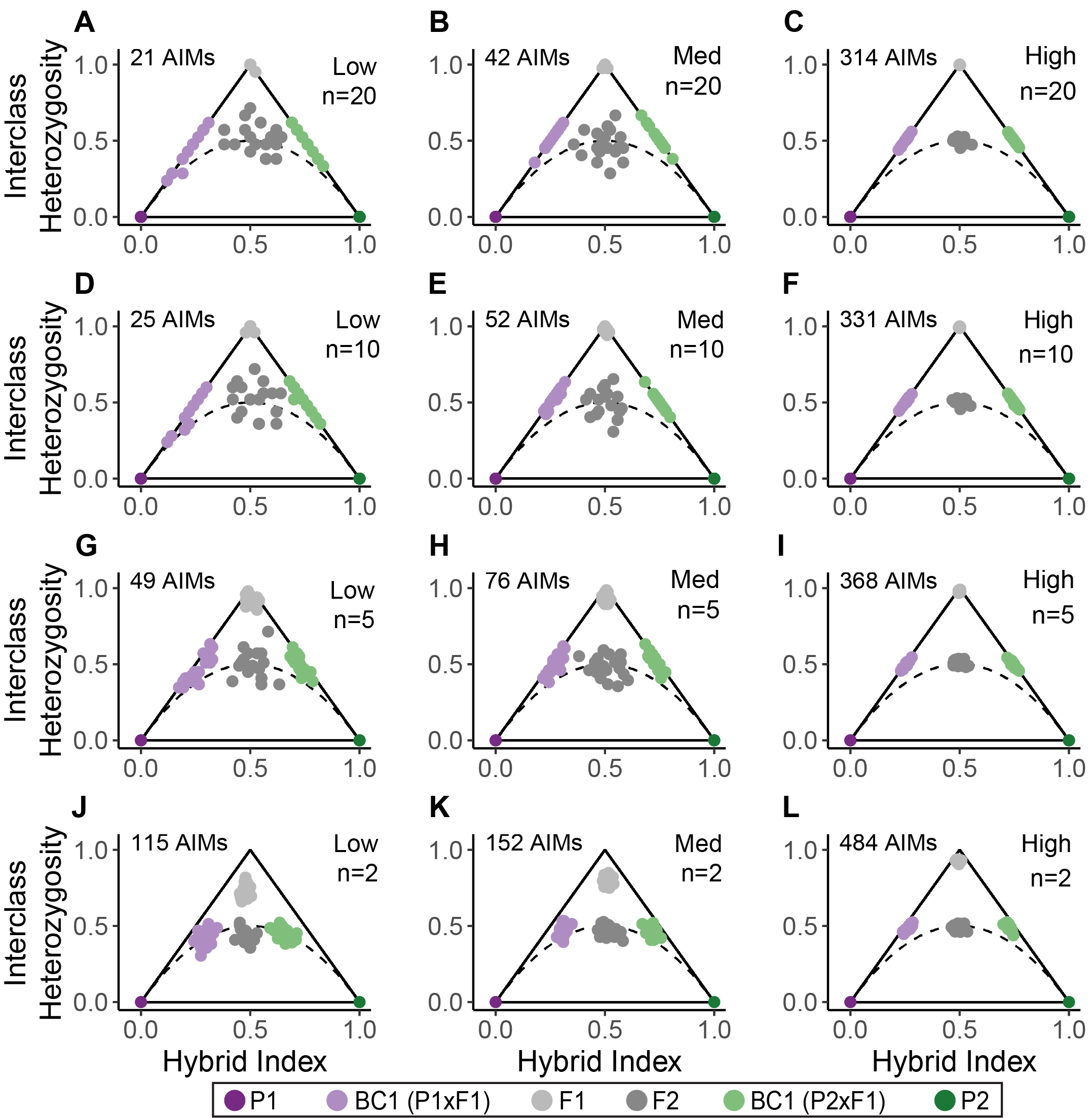


**Figure S5.** Triangle plots for known hybrids and parentals based on random samples of 20 **(A-C)**, 10 **(D-F)**, 5 **(G-I)**, or 2 **(J-L)** individuals (n) from each parental population. Sites that passed δ=1 in each sample were used as AIMs. The left column shows the simulation with low differentiation, the center column shows the simulation with medium differentiation, and the right column shows the simulation with high differentiation. Solid black lines indicate the possible space on a triangle plot, and the dotted black curve indicates the space below which individuals cannot occur, assuming Hardy-Weinberg Equilibrium.


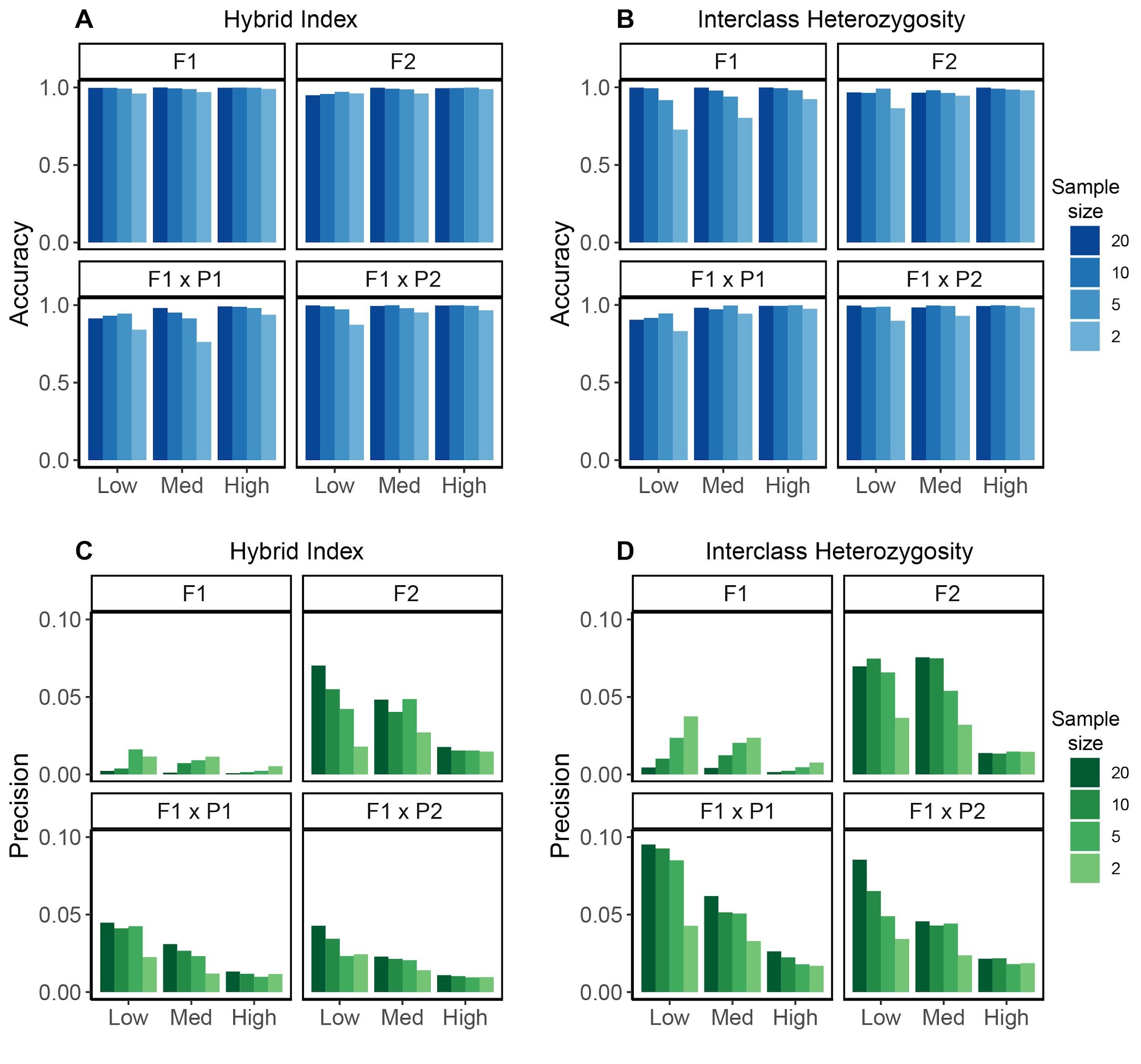


**Figure S6.** Accuracy **(A&B)** and precision **(C&D)** of hybrid index **(A&C)** and interclass heterozygosity **(B&D)** estimates based on random samples of 20, 10, 5, or 2 individuals from each parental population. Sites that passed δ=1 in each sample were used as AIMs. Each simulation (low, medium, high) is shown on the x-axis. Accuracy and precision was measured for 20 individuals from each of the four hybrid classes (F1, F2, and the two first generation backcrosses) separately. Accuracy is reported as a percent, with 1 indicating 100% accuracy. Precision is reported as the average Euclidean distance of each observation within a class from the average of that class, such that smaller values indicate higher precision.


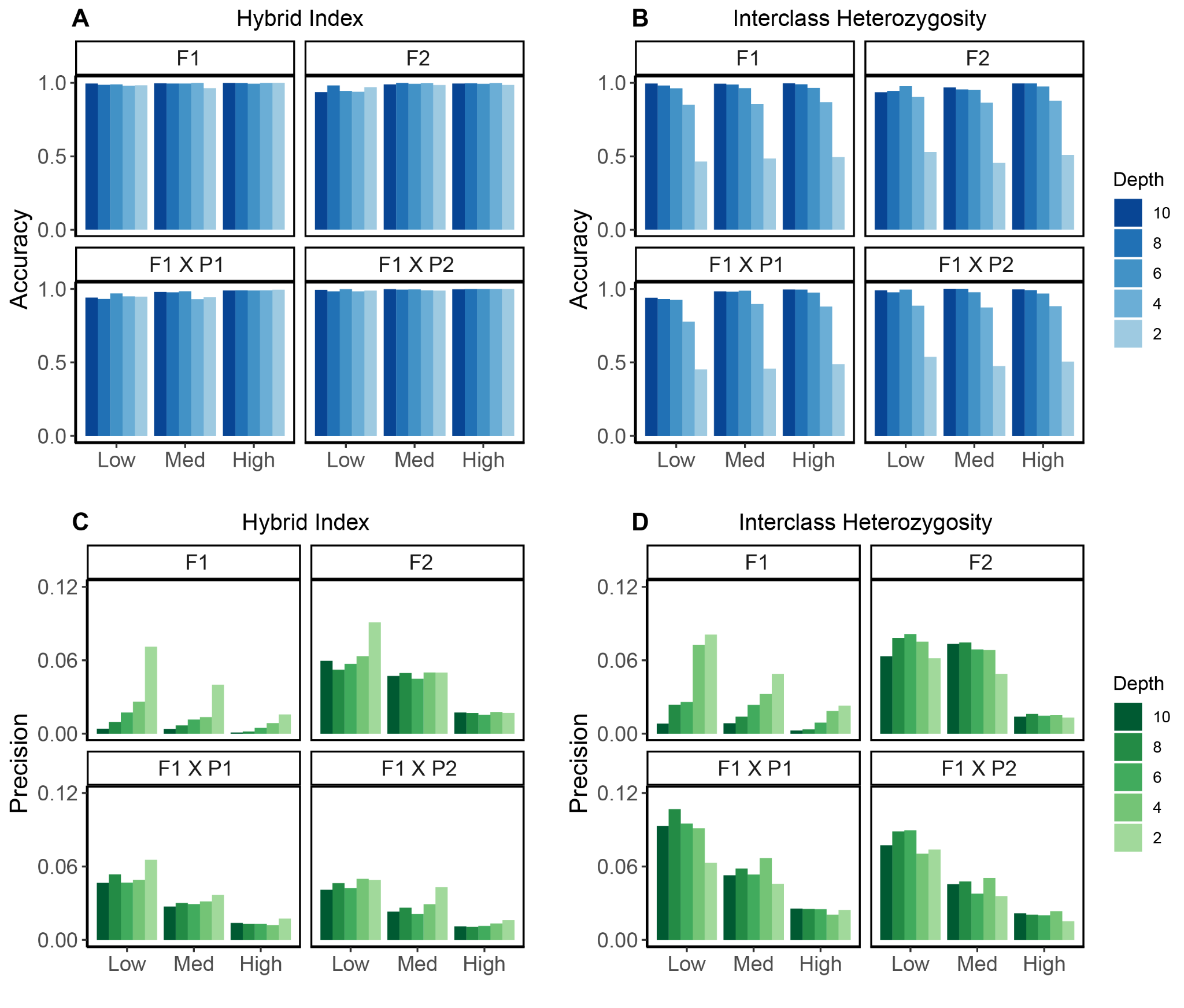


**Figure S7.** Accuracy **(A&B)** and precision **(C&D)** of hybrid index **(A&C)** and interclass heterozygosity **(B&D)** estimates based on simulated genotype depths of 10, 8, 6, 4, and 2. After recoding genotypes based on the observed depth, AIMs (δ=1) were called using a sample of 20 parentals. Each simulation (low, medium, high) is shown on the x-axis. Accuracy and precision was measured for 20 individuals from each of the four hybrid classes (F1, F2, and the two first generation backcrosses) separately. Accuracy is reported as a percent, with 1 indicating 100% accuracy. Precision is reported as the average Euclidean distance of each observation within a class from the average of that class, such that smaller values indicate higher precision.


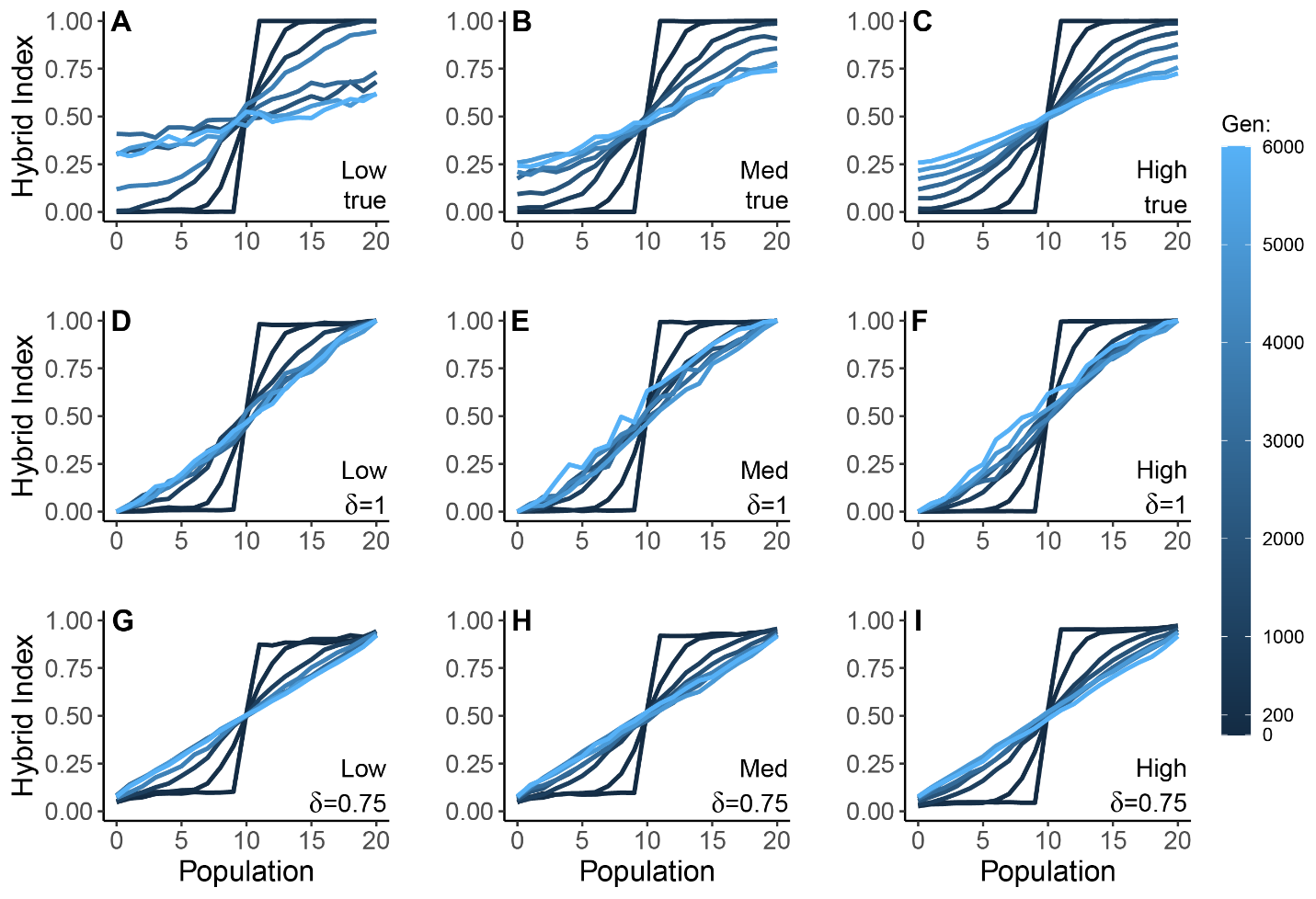


**Figure S8.** Hybrid index clines for true AIMs **(A-C)**, fixed differences identified within each sampled generation **(D-F)**, and AIMs that passed δ = 0.75 within each sampled generation **(G-I)**. The left column shows the simulation with low differentiation, the center column shows the simulation with medium differentiation, and the right column shows the simulation with high differentiation. Colors indicate the number of generations after initial contact, with generation 0 defined as the first generation in which all populations had at least 50 individuals during Phase III of the simulations.


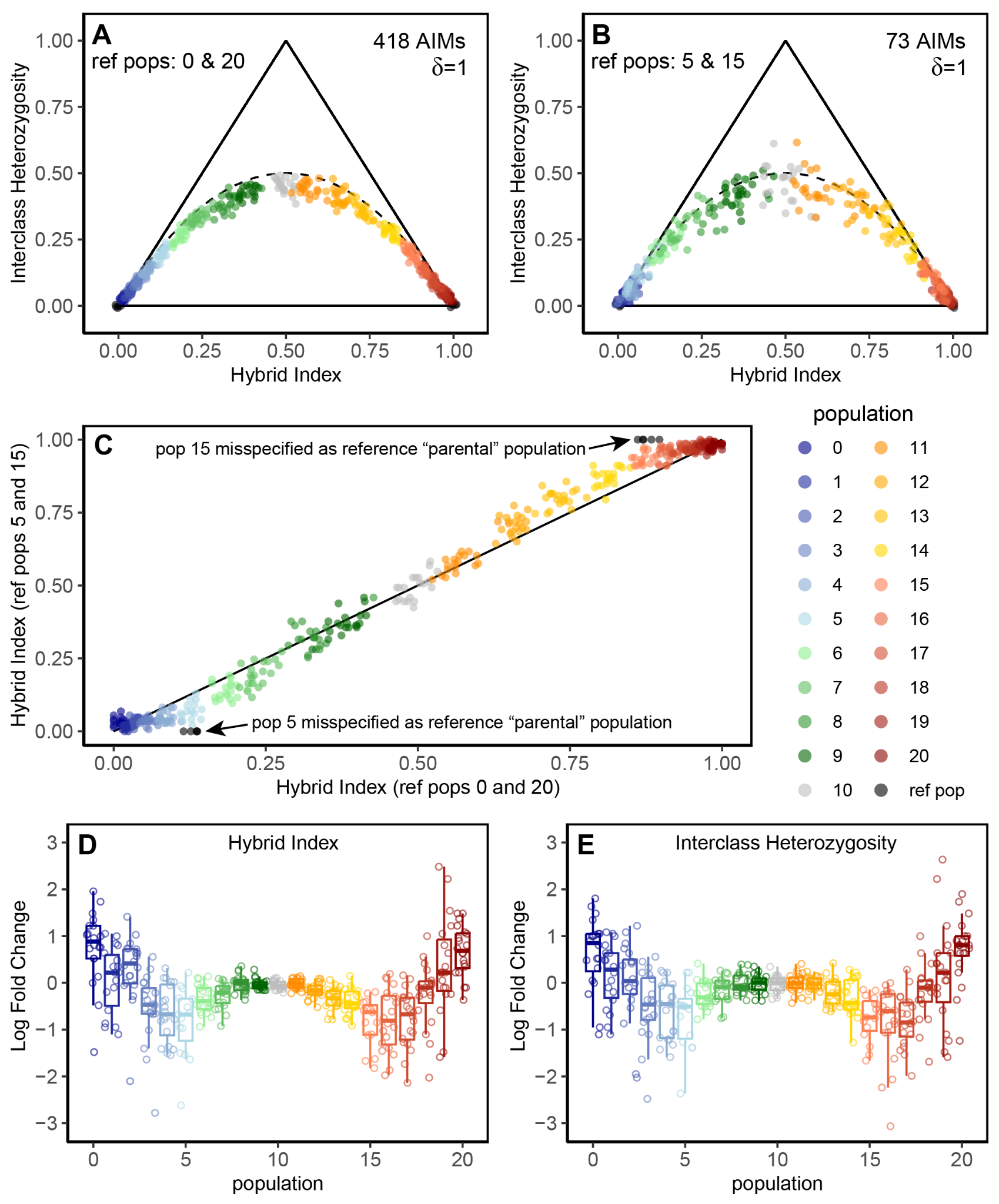


**Figure S9.** Hybrid index and interclass heterozygosity estimates for generation 1,000 of the high divergence simulation showing the effects of using misassigned parental individuals for identifying AIMs. **(A)** Triangle plot using five individuals from the correct (p0 and p20) parental groups. **(B)** Triangle plot using five individuals from misassigned (p5 and p15) parental groups. **(C)** Hybrid index estimates using AIMs identified with correct parental groups compared to those identified from misassigned parental groups. Log fold change in hybrid index **(D)** and interclass heterozygosity **(E)** estimates when using misassigned parental groups in relation to known values of hybrid index and interclass heterozygosity. Negative values represent shifts towards the nearest parental population and positive values represent shifts away from the nearest parental population.


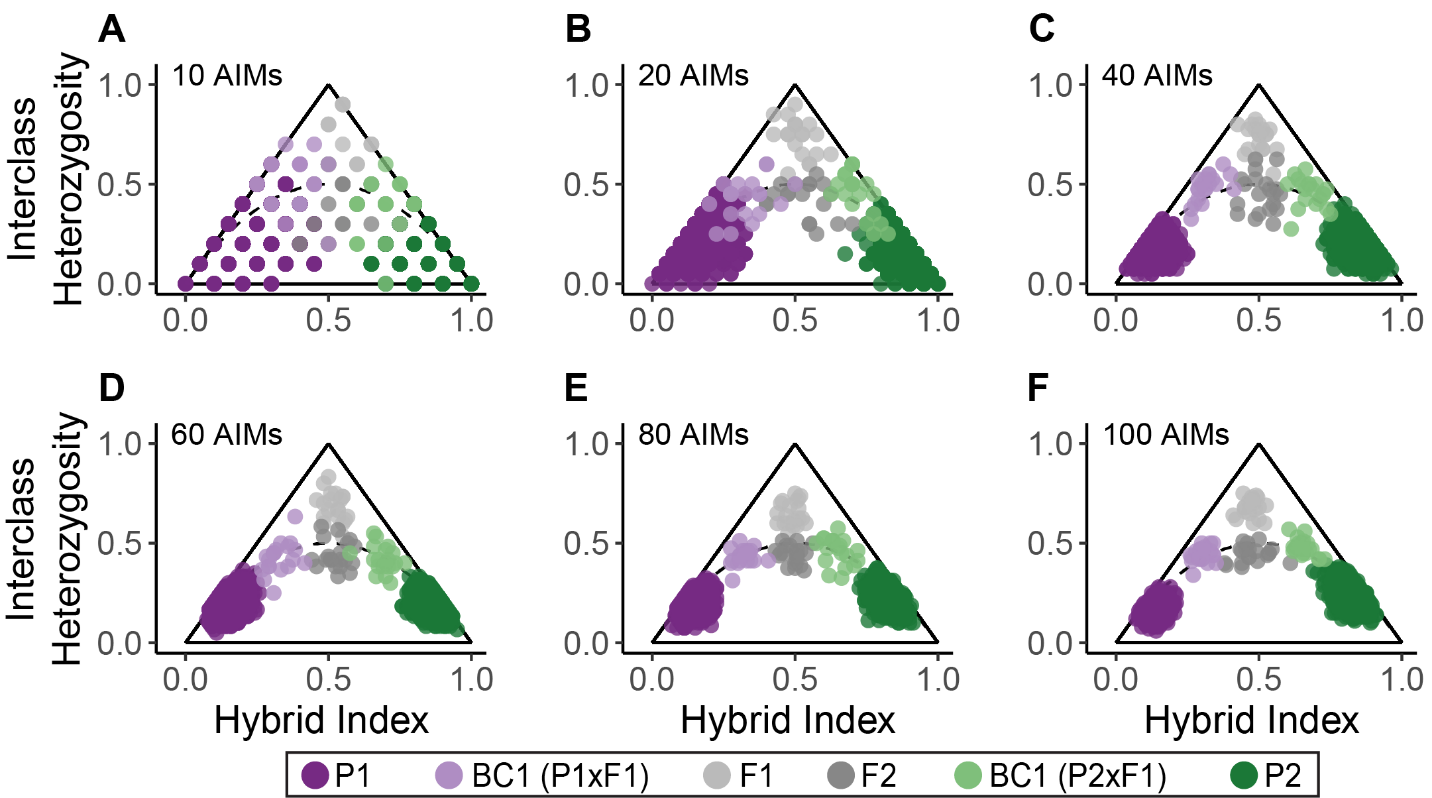


**Figure S10.** Triangle plots for known hybrids and parentals when divergence is low and few AIMs are identified. All plots are based on AIMs identified with the δ=0.5 threshold in the low divergence simulation. Of the AIMs identified with the δ=0.5 threshold (n=315), we randomly downsampled 10 **(A)**, 20 **(B)**, 40 **(C)**, 60, **(D)**, 80 **(E)**, and 100 **(F)** AIMs. Sampling AIMs in this way essentially models sequencing effort, and serves as a demonstration of the number of AIMs needed to accurately identify hybrid classes when parental divergence is low.


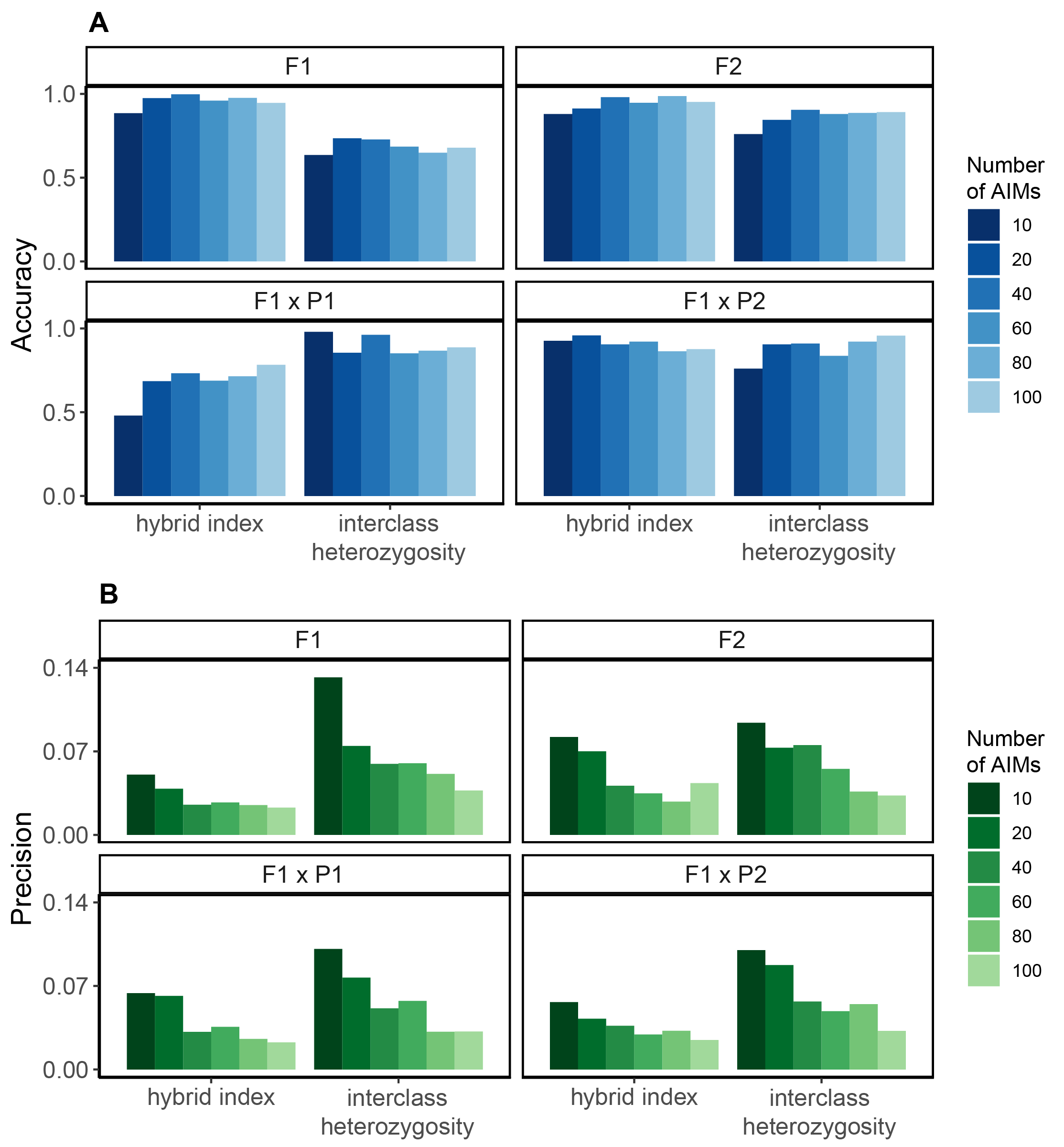


**Figure S11.** Accuracy **(A)** and precision **(B)** of hybrid index and interclass heterozygosity estimates when divergence is low and few AIMs are identified. AIMs were identified with the δ=0.5 threshold in the low divergence simulation, and were randomly downsampled to 10, 20, 40, 60, 80, and 100 AIMs. Accuracy and precision of the estimates was measured for 20 individuals from each of the four hybrid classes (F1, F2, and the two first generation backcrosses) separately. Accuracy is reported as a percent, with 1 indicating 100% accuracy. Precision is reported as the average Euclidean distance of each observation within a class from the average of that class, such that smaller values indicate higher precision.


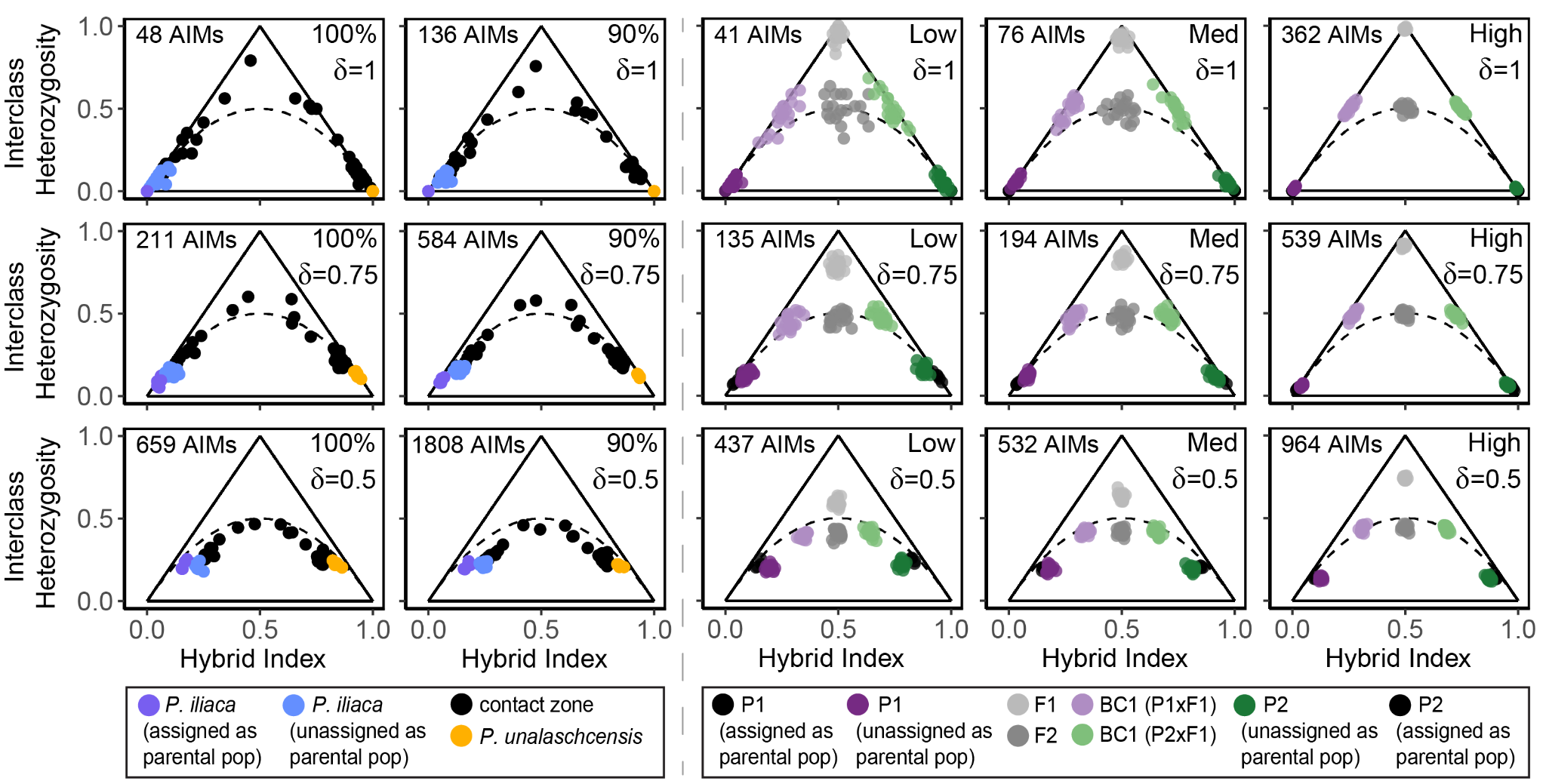


**Fig S12.** Triangle plots for empirical (*Passerella*) and simulated data. From left to right, the columns show triangle plots for the 100% complete *Passerella* dataset, 90% complete *Paserella* dataset, simulated low divergence, simulated medium divergence, and simulated high divergence. For each dataset, three allele frequency difference (δ) thresholds were used, from top to bottom, δ=1, δ=0.75, and δ=0.5. For the empirical data, even parental sample sizes (N=5) were used, and the remaining thirteen allopatric *P. iliaca* were left in the dataset unassigned as a parental group. For the simulated data, five parental individuals were assigned to each parental group, and twenty were included in the dataset but unassigned as the parental group used for identifying AIMs.

**Table S1.** Main functions of the R package *triangulaR*.

| Function | Description | Input | Output |
| --- | --- | --- | --- |
| alleleFreqDiff() | Identify AIMs from a SNP dataset. Implemented by filtering for SNPs that pass a given allele frequency difference threshold (δ) | vcfR object; popmap* | vcfR object with AIM genotypes |
| hybridIndex() | Calculate hybrid index and interclass heterozygosity for each individual | vcfR object of AIMs; popmap | R dataframe |
| triangle.plot() | Plot hybrid index and interclass heterozygosity of each individual on a triangle plot | R dataframe (from hybridIndex() function) | Triangle plot |
| missing.plot() | Color individuals on triangle plot by percent missing data | R dataframe (from hybridIndex() function) | Triangle plot |
| AIMnames() | Get the names of the SNPs that pass a given allele frequency difference threshold (δ) | vcfRobject; popmap | vector of SNP names |
| specFreqDiff() | Plot the distribution of allele frequency differences between the parental populations across all SNPs | vcfR object; popmap | R dataframe |
| aimFreqDist() | Visualize the distribution of allele frequencies of AIMs in each parental population | vcfR object; popmap | R dataframe |

*R dataframe with individual IDs in the first column and population assignments in the second column

**Table S2.** Descriptive statistics about the true allele frequencies of AIMs (δ=1) identified based on 200 random samples (n) of 20, 10, 5, and 2 parental individuals. “True δ<1” refers to the percent of AIMs that have δ=1 in the sample but δ<1 between the full populations. “True δ<0.95” refers to the percent of AIMs that have δ=1 in the sample but δ<0.95 between the full populations. Accuracy is defined as the average absolute difference between the expected value (true δ) and the observed value in the sample, divided by the expected value. We subtract that value from 1 to report the percent accuracy. “0.05 Quantile” indicates the value *x* for which 95% of identified AIMs have a true δ greater than *x*. Likewise “0.1 Quantile” and “0.25 Quantile” show this value at the 90% and 75% cutoffs, respectively.

| Sim | n | True δ<1 | True δ<0.95 | Accuracy | 0.05 Quantile | 0.10 Quantile | 0.25 Quantile |
| --- | --- | --- | --- | --- | --- | --- | --- |
| low | 20 | 28.8% | 3.9% | 99.3% | 0.96 | 0.98 | 0.99 |
| low | 10 | 45.1% | 17.7% | 97.8% | 0.89 | 0.92 | 0.98 |
| low | 5 | 63.8% | 42.1% | 93.1% | 0.72 | 0.78 | 0.89 |
| low | 2 | 85.7% | 76.1% | 76.4% | 0.39 | 0.47 | 0.63 |
| med | 20 | 19.1% | 2.9% | 99.5% | 0.97 | 0.98 | 1.00 |
| med | 10 | 33.6% | 14.3% | 98.2% | 0.90 | 0.94 | 0.98 |
| med | 5 | 53.1% | 35.9% | 94.3% | 0.77 | 0.82 | 0.91 |
| med | 2 | 77.1% | 67.5% | 81.4% | 0.44 | 0.54 | 0.69 |
| high | 20 | 4.6% | 0.7% | 99.9% | 1.00 | 1.00 | 1.00 |
| high | 10 | 9.7% | 3.8% | 99.5% | 0.96 | 1.00 | 1.00 |
| high | 5 | 18.8% | 12.0% | 98.2% | 0.87 | 0.94 | 1.00 |
| high | 2 | 38.0% | 31.9% | 91.9% | 0.58 | 0.70 | 0.89 |

**Table S3.** Run times for *triangulaR* and *bgchm*. Code for both programs was executed using R version 4.0.3 in RStudio (2024.09.1) on a computer with an i7 Intel Chip, 4 cores, and 16GB of RAM.

|  | **160 individuals** | | **360 individuals** | |
| --- | --- | --- | --- | --- |
|  | **346 AIMs** | **2076 AIMs** | **346 AIMs** | **2076 AIMs** |
| ***triangulaR*** | 0.05 seconds | 0.13 seconds | 0.08 seconds | 0.23 seconds |
| ***bgchm*** | 1.4 hours | 8.8 hours | 3.7 hours | 23.6 hours |

**Table S4.** Specimen Appendix

| **Sampling group** | **Specimen reference** | **Locality** |
| --- | --- | --- |
| Parental *iliaca* | UAM:bird:20392 | Alaska: Seward Peninsula; Nome area |
| Parental *iliaca* | UAM:bird:27545 | Alaska: Interior; Twelvemile Summit area |
| Parental *iliaca* | UAM:bird:27546 | Alaska: Interior; Twelvemile Summit area |
| Parental *iliaca* | UAM:bird:30343 | Alaska: Interior; Angel Creek and Chena River |
| Parental *iliaca* | UAM:bird:34092 | Alaska: Brooks Range; Wiseman |
| Parental *iliaca* | UAM:bird:34093 | Alaska: Alaska Range; Raindeer Hill |
| Parental *iliaca* | UAM:bird:34094 | Alaska: Alaska Range; Broad Pass |
| Parental *iliaca* | UAM:bird:37204 | Alaska: Interior; Chatanika River, Kokomo Creek |
| Parental *iliaca* | UAM:bird:37207 | Alaska: Interior; Chatanika River, Alder Creek |
| Parental *iliaca* | UAM:bird:37208 | Alaska: Interior; Yukon-Tanana uplands, Mt. Ryan |
| Parental *iliaca* | UAM:bird:37209 | Alaska: Interior; Yukon-Tanana uplands, Mt. Ryan |
| Parental *iliaca* | UAM:bird:37210 | Alaska: Interior; Taylor HWY along Wade Creek |
| Parental *iliaca* | UAM:bird:41097 | Alaska: Alaska; Alaska Range; Isabel Pass area |
| Parental *iliaca* | UAM:bird:41098 | Alaska: Alaska; Alaska Range; Isabel Pass area |
| Parental *iliaca* | UAM:bird:42685 | Alaska: Alaska Range; Denali Highway, mi 87.7 |
| Parental *iliaca* | UAM:bird:42686 | Alaska: Alaska Range; Denali Highway, mi 87.7 |
| Parental *iliaca* | UAM:bird:42840 | Alaska: Talkeetna |
| Parental *iliaca* | UAM:bird:38815 | Alaska: Unalakleet, Nulato Hills |
| Parental *unalaschcensis* | UAM:bird:38937 | Alaska: Prince William Sound; Valdez Narrows |
| Parental *unalaschcensis* | UAM:bird:44466 | Alaska: Prince William Sound; Valdez Narrows |
| Parental *unalaschcensis* | UAM:bird:40396 | Alaska: Prince William Sound; Whittier |
| Parental *unalaschcensis* | UAM:bird:30340 | Alaska: Prince William Sound |
| Parental *unalaschcensis* | UAM:bird:30341 | Alaska: Prince William Sound |
| Parental *unalaschcensis* | UAM:bird:31497 | Alaska: Kenai Peninsula |
| Hybrid zone | UAM:bird:34095 | Alaska: Upper Cook Inlet; Anchorage area |
| Hybrid zone | UAM:bird:34096 | Alaska: Upper Cook Inlet; Anchorage area |
| Hybrid zone | UAM:bird:34097 | Alaska: Upper Cook Inlet; Anchorage area |
| Hybrid zone | UAM:bird:34098 | Alaska: Upper Cook Inlet; Anchorage area |
| Hybrid zone | UAM:bird:34099 | Alaska: Upper Cook Inlet; Chugach Mts. |
| Hybrid zone | UAM:bird:34100 | Alaska: Upper Cook Inlet; Chugach Mts. |
| Hybrid zone | UAM:bird:34101 | Alaska: Upper Cook Inlet; Anchorage area |
| Hybrid zone | UAM:bird:34102 | Alaska: Upper Cook Inlet; Anchorage area |
| Hybrid zone | UAM:bird:34110 | Alaska: Upper Cook Inlet; Chugach Mts. |
| Hybrid zone | UAM:bird:34528 | Alaska: Upper Cook Inlet; Matanuska R. |
| Hybrid zone | UAM:bird:36899 | Alaska: Upper Cook Inlet; Anchorage area |
| Hybrid zone | UAM:bird:36900 | Alaska: Upper Cook Inlet; Anchorage area |
| Hybrid zone | UAM:bird:36901 | Alaska: Upper Cook Inlet; Anchorage area |
| Hybrid zone | UAM:bird:36902 | Alaska: Upper Cook Inlet; Anchorage area |
| Hybrid zone | UAM:bird:36903 | Alaska: Upper Cook Inlet; Anchorage area |
| Hybrid zone | UAM:bird:38224 | Alaska: Upper Cook Inlet; Anchorage area |
| Hybrid zone | UAM:bird:38225 | Alaska: Upper Cook Inlet; Anchorage area |
| Hybrid zone | UAM:bird:38226 | Alaska: Upper Cook Inlet; Anchorage area |
| Hybrid zone | UAM:bird:38228 | Alaska: Upper Cook Inlet; Anchorage area |
| Hybrid zone | UAM:bird:38229 | Alaska: Upper Cook Inlet; Anchorage area |
| Hybrid zone | UAM:bird:38230 | Alaska: Upper Cook Inlet; Anchorage area |
| Hybrid zone | UAM:bird:39646 | Alaska: Upper Cook Inlet; Turnagain Arm |
| Hybrid zone | UAM:bird:40393 | Alaska: Upper Cook Inlet; Anchorage area |
| Hybrid zone | UAM:bird:40394 | Alaska: Upper Cook Inlet; Anchorage area |
| Hybrid zone | UAM:bird:40395 | Alaska: Upper Cook Inlet; Anchorage area |
| Hybrid zone | UAM:bird:40397 | Alaska: Upper Cook Inlet; Anchorage area |
| Hybrid zone | UAM:bird:40398 | Alaska: Upper Cook Inlet; Anchorage area |
| Hybrid zone | UAM:bird:40399 | Alaska: Upper Cook Inlet; Anchorage area |
| Hybrid zone | UAM:bird:40400 | Alaska: Upper Cook Inlet; Anchorage area |
| Hybrid zone | UAM:bird:40402 | Alaska: Upper Cook Inlet; Anchorage area |
| Hybrid zone | UAM:bird:40404 | Alaska: Upper Cook Inlet; Anchorage area |
| Hybrid zone | UAM:bird:40405 | Alaska: Upper Cook Inlet; Anchorage area |
| Hybrid zone | UAM:bird:40406 | Alaska: Upper Cook Inlet; Anchorage area |
| Hybrid zone | UAM:bird:40407 | Alaska: Upper Cook Inlet; Anchorage area |
| Hybrid zone | UAM:bird:40408 | Alaska: Upper Cook Inlet; Anchorage area |
| Hybrid zone | UAM:bird:41230 | Alaska: Upper Cook Inlet; Anchorage area |
| Hybrid zone | UAM:bird:41231 | Alaska: Upper Cook Inlet; Anchorage area |
| Hybrid zone | UAM:bird:41233 | Alaska: Upper Cook Inlet; Anchorage area |
